# The IclR-family transcriptional regulator XyrR controls flotation, motility, antibiotic production and virulence in *Serratia* sp. ATCC 39006

**DOI:** 10.3389/fmicb.2024.1500889

**Published:** 2025-01-15

**Authors:** Carlo Miguel Castro Sandoval, George P. C. Salmond

**Affiliations:** ^1^Department of Biochemistry, University of Cambridge, Cambridge, United Kingdom; ^2^Institute of Crop Science, University of the Philippines Los Baños, Los Baños, Laguna, Philippines

**Keywords:** gas vesicles, carbapenem, prodigiosin, xylonate, motility, transcriptional regulator, *Prodigiosinella*, *Serratia*

## Abstract

The opportunistic pathogen *Serratia* sp. ATCC 39006 (S39006) is a rod-shaped, motile, Gram-negative bacterium that produces a 𝛽-lactam antibiotic (a carbapenem) and a bioactive red-pigmented tripyrrole antibiotic, prodigiosin. It is also the only known enterobacterium that naturally produces intracellular gas vesicles (GVs), enabling cells to float in static water columns. Regulation of GVs and secondary metabolites in S39006 can be coordinated but such pleiotropy is still poorly understood. To uncover novel inputs to this complex regulatory network, we used transposon mutagenesis to identify a mutant with an insertion in an IclR-type transcriptional regulator gene. The *iclR* mutant showed diminished production of carbapenem, prodigiosin, GVs and cellulase. Furthermore, the mutant also showed increased swimming and swarming motilities but exhibited attenuated virulence *in planta* and ability to kill the nematode *C. elegans*. Using differential expression analysis of the intracellular proteomes of the wild type and *iclR* mutant, we confirmed that the mutation negatively impacted expression of the corresponding GV, carbapenem and prodigiosin gene clusters. In contrast, flagellar and chemotaxis proteins were overexpressed, consistent with the increased motility of the mutant. We also found that the proteins encoded by a putative *yagEF-yjhF* operon, involved in xylonate catabolism and transport, showed a 5- to 7-fold increase in expression. Finally, we show that IclR is a repressor of xylonate catabolism in S39006 and suggest that xylonate is potentially involved in controlling carbapenem and prodigiosin biosynthesis. Our results indicate that IclR is a global regulator that controls antibiotic biosynthesis, flotation through modulating GV assembly, and has pleiotropic impacts on the physiology and virulence of S39006. Based on these findings, we propose the designation of this IclR-family transcriptional regulator as XyrR (*Xy*lonate *r*esponse *R*egulator).

## Introduction

*Serratia* sp. ATCC 39006 (S39006) produces hollow, proteinaceous, intracellular gas vesicles that enable cells to float and colonize air-liquid interfaces (Ramsay et al., 2011; Monson et al., 2016; Tashiro et al., 2016). Recent phylogenetic studies have proposed reclassifying S39006 first as *Prodigiosinella confusarubida* (Duprey et al., 2019) and more recently as *Prodigiosinella aquatilis* ATCC 39006 (Hugouvieux-Cotte-Pattat et al., 2024). However, for consistency with existing literature, we will continue to refer to this strain as S39006 in this study. Flotation provides S39006 with a passive and energy-efficient form of movement that is independent of flagella. This strain also produces two key secondary metabolites, a red pigmented antibiotic, prodigiosin and a 𝛽-lactam antibiotic 1-carbapen-2-em-3-carboxylic acid (a carbapenem; Parker et al., 1982; Thomson et al., 1997). S39006 is a phytopathogen that causes soft rot in potato by secreting the plant cell wall degrading enzymes (PCWDEs) cellulase and pectate lyase (Fineran et al., 2007) and can kill the nematode *C. elegans* (Coulthurst et al., 2004). Control of secondary metabolite biosynthesis, motility and virulence in S39006 is modulated by quorum sensing (QS) control through bioactive transcriptional regulation, two component systems, transport proteins and post-transcriptional regulators (Thomson et al., 1997, 2000; Slater et al., 2003; Fineran et al., 2005a; Fineran et al., 2005b; Williamson et al., 2008; Wilf et al., 2011; Wilf and Salmond, 2012). However, fewer regulators of gas vesicle biogenesis have been reported thus far.

The regulation of gas vesicle production in S39006 has been studied for over a decade. Quorum sensing, the Rsm system (a homolog of the *E. coli* Csr system) and the cognate regulatory proteins encoded in the GV cluster (GvrA, GvrB and GvrC) were among the initially defined modulators of flotation in S39006 (Ramsay et al., 2011). Random transposon mutagenesis screens have further identified regulatory inputs to this complex network. Several proteins with a wide range of functions have been linked to gas vesicle production including PigP, RbsR, TopA, FloR, TrkH, RpoN and DksA (Fineran et al., 2005a; Lee et al., 2017; Lee, 2018; Quintero-Yanes et al., 2019; Quintero-Yanes et al., 2020; Hill, 2021). Moreover, environmental cues such as oxygen limitation and potassium levels have been shown to affect the production of gas vesicles. The expression of the GV cluster is significantly upregulated in microaerophilic conditions and this response is postulated to be transduced via the *gvrA-gvrC* operon (Ramsay et al., 2011). A mutation in *trkH* (encoding a low-affinity potassium transporter) resulted in the hyperproduction of gas vesicles in S39006 (Quintero-Yanes et al., 2019). In contrast, GV biogenesis, flotation, and expression of the *gvpA1-gvpY* operon were reduced in wild type S39006 at high potassium concentrations. These findings confirmed that potassium, imported by the cell via TrkH, is a key environmental signal controlling cell flotation in S39006. Another mechanism in which S39006 senses environmental cues to regulate flotation is via the alternative sigma factor RpoN (σ^54^) although the exact signal is still unknown. A mutant with a transposon insertion in *rpoN* lost the ability to produce gas vesicles (Hill, 2021). Furthermore, fusion assays revealed that transcription of the *gvpA1-gvpY* operon significantly decreased while *gvrA-gvrC* expression was unchanged compared to the wild type. This indicated that σ^54^ acts directly on transcription the *gvpA1-gvpY* operon to control its transcription. It has been proposed that σ^54^ and RNAP bind the −12 and − 24 sites upstream of *gvpA1-gvpY*, which is then converted to an open complex by the binding of GvrA.

Master transcriptional regulators have also been implicated in the control of gas vesicle production. The global regulator FloR (a DeoR family transcription factor) controls GV and secondary metabolite production, motility and virulence in S39006 (Quintero-Yanes et al., 2020). A quantitative proteomic analysis of the *floR* transposon mutant indicated that FloR operates as a physiological master regulator by controlling expression of multiple pleiotropic regulators including Rap, RsmA, RpoS, PstS, PigT and PigU (Quintero-Yanes et al., 2020). PigP, originally discovered as master regulator of secondary metabolite production, was only recently investigated for its regulatory role in gas vesicle biogenesis (Fineran et al., 2005b; Lee, 2018). Transcriptional reporter fusions in the initial gene of each operon in the GV cluster revealed that disruption of PigP attenuated the expression *gvpA1-gvpY* but not *gvrA-gvrC* (Lee, 2018). The ribose operon repressor RbsR, a LacI family transcriptional regulator, has been linked to the activation of the gas vesicle production (Lee et al., 2017). Although gel shift assays did not provide evidence that RbsR altered transcription of the GV cluster by binding to its predicted site upstream of *gvrA-gvrC*, the study was able to show that S39006 produced significantly fewer gas vesicles on ribose minimal media (Lee et al., 2017). RbsR, together with RsmA, link gas vesicle regulation to carbon metabolism.

Here we report an IclR family transcriptional regulator controlling multiple phenotypes in S39006. Disruption of *iclR* in S39006 reduced the flotation ability, virulence and secondary metabolite production but enhanced the bacterium’s motility. We also show the IclR protein represses expression of xylonate catabolic genes by binding its promoter.

## Materials and methods

### Bacterial strains, bacteriophage and culture conditions

All bacterial strains and the general transducing phage used in the study are listed in Table 1. *E. coli* strains were grown at 37°C while *Serratia* and *Pectobacterium* strains were grown at 30°C in Lysogeny Broth (LB). Overnight liquid cultures were grown in 5 mL LB in a 30 mL plastic universal tube, inoculated with a single bacterial colony and aerated on a rotary wheel for 14–16 h. Bacterial growth was determined by measuring the optical density of the liquid culture at 600 nm wavelength using a Unicam Heλios spectrophotometer (Thermo Scientific™).

**Table 1:** Bacterial strains and phage used in the study.

BACTERIA	Genotype or description	Reference
*E. coli*		
DH5α	F^−^ λ^−^ *ilvG*^−^ *rfb-*50 *rph*-1	Blattner et al. (1997)
*β*2163	F^−^ *ompT gal dcm lon hsdS_B_* (*r_B_*^−^*m_B_*^−^) *λ*(DE3 [*lacI lacUV5*-*T7p07 ind1 sam7 nin5*]) [*malB*^+^]_K-12_(λ^S^)	Studier and Moffatt (1986)
ESS	*β*-lactam super sensitive strain	Bainton et al. (1992)
OP50	Regular feeder strain for *C. elegans*	Brenner (1973)
*Serratia* sp.		
S39006 LacA	*Lac*^−^ derivative of *Serratia* sp. ATCC 39006	Thomson et al. (2000)
NWA19	*lacA*, *ΔpigC*, constructed by marker exchange using pNRW54	Ramsay et al. (2011)
GPA1	*lacA*, *gvpA1*::mini-Tn5*uidA*, Cm^R^	Ramsay et al. (2011)
GRA	*lacA*, *gvrA*::mini-Tn5*uidA*, Cm^R^	Ramsay et al. (2011)
MCA54	*carA*::mini-Tn5*lacZ1*, Kn^R^	Thomson et al. (2000)
CS109	*lacA*, *iclR*::mini-Tn5*lacZ1*, Kn^R^	This study
CS119	*lacA*, *gvpA1::*mini-Tn5*uidA*, *iclR*::mini-Tn5*lacZ1*, Cm^R^, Kn^R^	This study
CS129	*lacA*, *gvrA::*mini-Tn5*uidA*, *iclR*::mini-Tn5*lacZ1*, Cm^R^, Kn^R^	This study
Phage		
ØOT8	*Serratia* general transducing phage	Evans et al. (2010)

### Transposon mutagenesis and screening

Random transposon mutagenesis was carried out using a plasmid-transposon (plasposon) hybrid system, pKRCPN1, as previously described (Monson et al., 2015; Lee et al., 2017). The plasmid-transposon hybrid vector pKRCPN1 was delivered into NWA19 recipient cells by conjugation using the DAPA auxotroph *E. coli β*2163 as donor strain. Conjugations were performed by mixing overnight cultures that had been normalized to an OD_600_ of 1.0 in a 1:2 or 1:3 donor to recipient ratio. Putative transconjugants were plated onto selective agar (LB + Kn). The absence of DAPA in the plates allows counterselection of the donor while kanamycin ensured selection of successful NWA19 transconjugants. Resuspended cells from donor-and recipient-only spots were also plated out onto selective agar as controls.

Putative transconjugants were visually screened for any notable difference in colony opacity, compared to NWA19, as a proxy for gas vesicle production. Colonies that were noticeably more translucent, slightly less opaque, hyper opaque or that had an unusual colony morphology were selected putative gas vesicle mutants. The putative mutants were passaged thrice on selective agar to confirm the observed colony phenotypes. The transposon insertion location in the mutants was determined by random primed PCR (RP-PCR) (Jacobs et al., 2003) and subsequent DNA sequencing across the transposon junction using oligos oPF109 and oMAMV2. Prior to characterization, the transposon insertion was transferred into a clean S39006 LacA background by transduction using the generalized transducing phage ϕOT8 (Evans et al., 2010).

### Phenotypic assays

Phenotypic plate assays for carbapenem, cellulase, and swimming and swarming motilities were performed as described previously (Slater et al., 2003; Andro et al., 1984; Lee et al., 2017). Overnight cultures of the test strains were normalized to an OD_600_ of 1.0 before spotting 10 μL (or 5 μL for swimming and swarming assays) onto appropriate agar plates. Indicator plates for carbapenem production were prepared using LB agar overlaid with 0.75% top agar seeded with *E. coli* ESS. Antibiotic production was indicated by inhibition zones around test colonies (Slater et al., 2003). Carboxymethylcellulose plates for cellulase assays were developed by flooding with 0.2% (w/v) Congo red for 20 min, bleached with 1 M NaCl then stained with 1 M HCl for 5 min (Andro et al., 1984). Cellulase activity was indicated by a halo surrounding the test strain. Swimming and swarming plate assays were assessed visually as previously described (Lee et al., 2017).

### Virulence assays

Potato rotting assays were performed as described by Walker et al. (1994) with some modifications. Maris Piper potatoes were surface sterilized by dipping the tubers in 1% (w/v) Virkon™ solution for 10 min, rinsed with sterile deionized water and air-dried at RT. Overnight cultures of the test strains were normalized to contain approximately 10^7^ CFU/mL based on the OD_600_. Using a sterile 200-μL pipette tip, holes of the same depth were created on opposite sides of a potato tuber and inoculated with 10 μL (10^5^ cells) of the WT and mutant strains. The infection sites were sealed with sterile vacuum grease and the tuber was wrapped in several layers of wet paper towel then finally with cling film. The inoculated tubers were incubated at 30°C for 5 days to allow infection. After incubation, the tubers were cross sectioned, and the rotten tissue was scraped off and weighed. To determine the bacterial load, 0.1 g of rotten tissue samples were resuspended in 1 mL of 1X M9 solution by vortexing. A 100 μL aliquot of the resuspended cells were serially diluted and plated onto LB plates to determine the number of viable cells. Viable cell count for each strain was expressed as CFU per 0.1 g of rotten tissue.

*C. elegans* killing assays were performed based on the method described by Kurz et al. (2003), using the DH26 strain. The nematodes were maintained on Nematode Growth Medium (NGM) agar covered with an *E. coli* OP50 lawn. Prior to each killing assay, the nematodes were synchronized to ensure all worms used were at the same development stage. Fifty (50) L4 stage nematodes were used for each test strain in the killing assay. Ten nematodes were placed onto each NGM plate containing a 50 μL spot of the test strain (grown overnight at 30°C prior to the worm transfer). The plates were then incubated at 25°C and scored for dead nematodes every 24 h until the population was eradicated. Nematodes that no longer responded to touch were considered dead and live worms were transferred onto fresh plates every day.

### *β*-Glucuronidase and *β*-galactosidase reporter assays

*β*-glucuronidase (*β*-glu) activity was determined throughout growth as described by Ramsay et al. (2011). Aliquots (100 μL) from growth experiments of S39006 strains containing *uidA* gene fusions were taken and frozen immediately at −80°C. The samples were thawed at room temperature and 10 μL of each was transferred to 96-well microtiter plates. The samples were mixed with 100 μL of PBS containing 20 mg/mL lysozyme and 250 μg/mL 4′-methylumbelliferyl-*β*-D-glucuronide (MUG). Fluorescence emission was measured using the Gemini XPS plate reader (Molecular Devices) under the following settings: excitation 360 nm, emission 450 nm, cut-off 435 nm, 8 reads per well measured every 30 s for 30 min. The *β*-glu activity of each sample was expressed as Relative Fluorescence Units (RFU)/OD_600_.

The *β*-galactosidase (*β*-gal) activity of *E. coli* DH5α strains carrying *lacZ* promoter fusion plasmids was measured in the same manner except the substrate used was 4′-methylumbelliferyl-*β*-D-galactoside.

### Microscopy

For Phase Contrast Microscopy (PCM), wet mounts of liquid culture or colonies resuspended in LB were used for phase contrast imaging. Samples were visualized using an Olympus BX-51 microscope with 100X oil immersion lens and images were captured with QICAM Fast 1,394 digital camera.

Transmission Electron Microscopy (TEM) was performed at the Cambridge Advanced Imaging Center, University of Cambridge. Bacterial samples were attached to a carbon-coated glow discharge grid for 10 min and washed thrice with dH_2_O. The cells were stained with 2% phosphotungstic acid (pH 7.0) for 5 min and viewed under a FEI Tecnai G2 TEM.

### Construction of plasmids

Plasmids pQE80-*iclR,* pQE80Cm*-iclR* and pBAD30-*iclR* for genetic complementation were constructed using standard restriction cloning protocols. Oligonucleotides used in PCR amplification and plasmids used in this study are listed in Tables 2, 3. The coding sequence of *iclR* was amplified with primer pairs oCMCS14/oCMCS15 and oCMCS16/oCMCS15 for cloning into pQE80-*oriT* and pBAD30, respectively. To construct pQE80-*iclR* and pQE80Cm-*iclR*, the PCR product was digested with *Bam*HI (NEB) and *Hin*dIII (NEB) and ligated into compatibly digested pQE80-*oriT* or pQE80-*oriT*-Cm with T4 DNA ligase (Thermo Scientific), according to manufacturer’s instructions. Plasmid pBAD30-*iclR* was likewise constructed as described above except the PCR amplicon and pBAD30 were digested with *Sph*I and *Hin*dIII. The promoter fusion plasmid pRW50-*yag*E_prom_ was constructed by Gibson cloning using a prepared reaction mix (Supplementary Table 1). The plasmid backbone was amplified by long PCR using PrimeSTAR® GXL DNA Polymerase (Takara) and the 150 bp fragment containing the *yagE* promoter region was amplified using Phusion DNA polymerase. Equimolar amounts of the DNA fragments to be assembled were mixed used for the reaction. Gibson assembly was carried out by mixing 15 μL of the reaction buffer with 5 μL of DNA fragment mixture and incubated at 50°C for 1 h. All plasmids were subjected to sequencing (Eurofins Genomics or DNA Core Sequencing Facility, Department of Biochemistry, University of Cambridge) to confirm the sequence.

**Table 2:** Oligonucleotides used in the study.

Name	Sequence (5′-3′)	Description/Reference
Random primed PCR		
oPF106	GACCACACGTCGACTAGTGCNNNNNNNNNNAGAG	Random primer for RP-PCR (Fineran et al., 2007)
oPF107	GACCACACGTCGACTAGTGCNNNNNNNNNNACGCC	Random primer for RP-PCR (Fineran et al., 2007)
oPF108	GACCACACGTCGACTAGTGCNNNNNNNNNNGATAC	Random primer for RP-PCR (Fineran et al., 2007)
oPF109	GACCACACGTCGACTAGTGC	Adapter-specific primer for RP-PCR (Fineran et al., 2007)
oMAMV2	GCATAAAGCTTGCTCAATCAATCAC	Adapter-specific primer for RP-PCR (Matilla et al., 2012)
Cloning		
oCMCS10	AAGCTTTTGTCAGTGCGCAA	Forward primer for amplifying the pRW50 backbone for Gibson assembly
oCMCS11	GGCTGCAGGTCGACGGATCC	Reverse primer for amplifying the pRW50 backbone for Gibson assembly
oCMCS14	TAAGCAGGATCCATGCCAATCATTCAATCGGT	Forward primer for cloning of *iclR* into pQE80-*oriT* or pQE80-Cm (restriction site - *Bam*HI)
oCMCS15	TAAGCAAAGCTTTCAACCGTTAGCGTCATTGT	Reverse primer for cloning of *iclR* into pQE80-*oriT* or pQE80-Cm or pBAD30 (restriction site - *Hind*III)
oCMCS16	TAAGCAGCATGCATGCCAATCATTCAATCGGT	Forward primer for cloning of *iclR* into pBAD30 (restriction site - *SphI*)
oCMCS17	CGTCGACCTGCAGCCAAATTGCAGTGTTAGTATCA	Forward primer for cloning the 150-bp *yagE* promoter fragment into pRW50 using Gibson assembly (pRW50 amplified with oCMCS10 and oCMCS11)
oCMCS18	CGCACTGACAAAAGCTTTTAACCGCTCCTTACATCAT	Reverse primer for cloning the 150-bp *yagE* promoter fragment into pRW50 using Gibson assembly (pRW50 amplified with oCMCS10 and oCMCS11)
Electric mobility shift assay		
Cy5-LUEGO	GTGCCCTGGTCTGG	Cyanine5-labeled universal electrophoretic gel shift oligonucleotide (LUEGO) (Jullien and Herman, 2011)
oCMCS21	CACCACAAATTGCAGTGTTAGTATCATTTCGTTAATGTAGAACTTAGTTCTATAATATAAAACAAAATATCAATGAAGATCGACTGACACATATCGATTGTTGTGA	Short oligonucleotide for LUEGO probe containing the XynR box located in the *yagE* promoter region
oCMCS22	TCACAACAATCGATATGTGTCAGTCGATCTTCATTGATATTTTGTTTTATATTATAGAACTAAGTTCTACATTAACGAAATGATACTAACACTGCAATTTGTGGTGCCAGACCAGGGCAC	Long oligonucleotide for LUEGO probe containing the XynR box located in the *yagE* promoter region

**Table 3:** Plasmids used in the study.

Name	Description/Genotype	Reference
pKRCPN1	*tetA, tnp, ‘lacZ, oriR6K, aph*, Km^R^	Monson et al. (2015)
pQE80-*oriT*	pQE80L carrying the RK2 origin of transfer cloned as an *Nde*I fragment, Ap^R^	Ramsay et al. (2011)
pQE80-Cm	Derivative of pQE80-*oriT* with the *bla* casette replaced with cat casette via Gibson assembly	This study
pBAD30	Expression vector with *araBAD* promoter; derivative of pACYC-184; Cm^R^	Guzman et al. (1995)
pRW50	Promoterless *lacZ* fusion plasmid, RK2 replicon, Tc^R^	Lodge et al. (1992)
pQE80*-iclR*	pQE80-*oriT* containing the ORF of S39006 *iclR* as a *Bam*HI and *Hind*III fragment, Ap^R^	This study
pQE80-Cm-*iclR*	pQE80-Cm containing the ORF of S39006 *iclR* as a *Bam*HI and *Hind*III fragment, Cm^R^	This study
pBAD30-*iclR*	pBAD30 containing the ORF of S39006 *iclR* as a *Bam*HI and *Sph*I fragment, Ap^R^	This study
pRW50-*yagE*_prom_	pRW50 derivative containing the S39006 *yagE* promoter constructed via Gibson assembly	This study

### Protein expression and purification

IclR was overexpressed and purified in S39006 to preserve the nativity of the protein. Plasmid pQE80-*iclR* was transferred into S39006 by conjugation using *E. coli β*2163. IclR expression was induced with 1 mM IPTG at mid-logarithmic phase of growth and the IclR protein was purified using Ni-NTA agarose (Qiagen) according to manufacturer’s instructions. The protein content of the purified sample was measured by Bradford assay using BSA as standard. The identity of the purified his-tagged protein was confirmed by SDS-PAGE and Western blot analysis against the 6xHis tag.

### Electrophoretic mobility shift assay

Preparation of DNA probes and EMSA analyses were carried out according to the Labeled Universal Electrophoretic Gel Shift Oligonucleotide (LUEGO) method with minor modifications (Jullien and Herman, 2011). The fluorescently labeled Cy5-LUEGO was used to generate the different probes together with the designed “short” and “long” oligonucleotides (Table 2). Labeled probes were prepared by mixing the oligonucleotides in a 5:5:1 (LUEGO:short:long) ratio and annealed using the Veriti Thermo Cycler (Applied Biosystems) with the following profile: 2 min at 95°C then a 100% ramp down to 70°C followed by slow cooling at 0.1% ramp rate to 18°C. The resulting probe was diluted to 5 nM and titrated with increasing concentrations of the purified test protein. The protein-probe mixture was incubated in 1X EMSA reaction buffer (10 mM Tris–HCl pH 8, 5 mM DTT, 50 mM KCl, 1 mM MgCl_2_, 10% (v/v) glycerol, 0.1% Triton X-100) for 30 min at 20°C to allow binding. The reaction mixture was loaded onto a native resolving gel and separated in 1X TGE (0.5 mM Tris–HCl pH 8, 19.2 mM glycine, 0.2 mM EDTA) at 100 V. Gels were imaged using the Bio-Rad ChemiDoc™ MP imaging system at the excitation wavelength of Cy5 (650 nm).

### Preparation of cell lysates, TMT-labeling and LC–MS/MS

The protocol for the preparation of cell lysates for proteomic analysis was largely as described by Dolan et al. (2020). Briefly, 25 mL of LB was inoculated with overnight cultures of the strain of interest to starting OD_600_ of 0.05. Strains were grown for 14 h in 250 mL flasks at 30°C and 215 rpm. These cultures were then normalized to an OD_600_ of 2.0 and 25 mL of cells was harvested by centrifugation (2,739 g, 10 min, 4°C). The cell pellets were resuspended in 500 μL lysis buffer (100 mM Tris–HCl, 50 mM NaCl, 10% (v/v) glycerol, 1 mM Tris (2-carboxyethyl) phosphine, 1 mM cOmplete Mini protease inhibitor cocktail (Roche), pH 7.5) followed by three rounds of sonication (3 × 10 s), with 30 s rest between each round. Samples were then centrifuged (21,130 g, 15 min, 4°C) to remove cell debris. Protein content was determined using a Bradford assay (Bio-Rad) and samples were normalized to 2 mg/mL. At this stage, an SDS-PAGE analysis of the protein extracts and a western blot for GvpC were performed as quality controls. Cell lysates containing 100 μg protein were then sent to the Cambridge Center for Proteomics (CCP) for quantitative proteomic analysis.

### Statistical analyses

The statistical significance of any differences seen in RFU or antibiotic production over the course of a growth curve was determined using a repeated measures ANOVA where *p*-values of less than 0.05 were considered significant. Differences in the weight of rotten tissues from potato tubers between test strains was evaluated using a paired t-test. Viable cell counts from rotten tissues were likewise compared using a paired *t*-test. Finally, differences in the virulence of *C. elegans* were assessed using a Mantel-Cox log-rank test. Asterisks in figure legends indicate *p*-values: *, *p* < 0.05; **, *p* < 0.01; ***, *p* < 0.001; ****, *p* < 0.0001; NS, not significant.

## Results

### Generation of random transposon mutant library

S39006 colonies are opaque due to the ability of gas vesicles to refract light. Therefore, changes in colony opacity can be used as a simple and rapid visual screen for gas vesicle regulatory mutants. However, the opacity of S39006 colonies can be confounded in certain mutants where production of the red pigment, prodigiosin, is also altered. To overcome this complication, transposon mutagenesis was carried out in the non-pigmented S39006 strain, NWA19. This strain has an in-frame deletion of *pigC* which encodes the condensing enzyme necessary for prodigiosin production. Random transposon mutagenesis was carried out by conjugation of the DAPA auxotroph *E. coli β*2163 harboring plasposon pKRCPN1 with NWA19 cells. The pKRCPN1 plasmid contains a Tn*5* transposon that is fused to a promoterless *lacZ* gene, an *oriR6K* replication origin and a kanamycin resistance cassette. In addition, the plasmid backbone contains a transposase gene to facilitate transposition and a tetracycline resistance cassette. Since the plasmid requires the *pir*-encoded *π* protein for replication which is present in the donor strain but not in NWA19, the Tn*5* transposon is forced to integrate randomly into the recipient’s chromosome. By screening transconjugants for kanamycin resistance and tetracycline sensitivity, successful integration of only the transposon and not the entire plasmid can be easily confirmed. Lastly, the absence of DAPA in the screening plates was used to select against *E. coli β*2163.

A total of 30,072 transposon mutants were screened from 25 independent conjugation patches. The insertion mutants were visually screened based on differences in colony opacity or morphology relative to the recipient strain NWA19 and were classified into four distinct categories: translucent, intermediate, bull’s-eye and hyper opaque (Hill, 2021). From this mutant bank, nine (9) novel gas vesicle regulatory mutants (*rbsB*, *orf1875*, *draG*, *cysB*, *sdhC*, *orf5465*, *ubiJ*, *orf6410*, and *fliD*) were identified. The corresponding transposon insertions were transferred into a LacA (wild-type) background prior to further characterization. Here, we present the characterization of a mutant, CS109, with a transposon inserted in *orf6410* which is predicted to encode an IclR-type transcriptional regulator.

### Sequence analysis and identification of *iclR*

**Figure 1:**
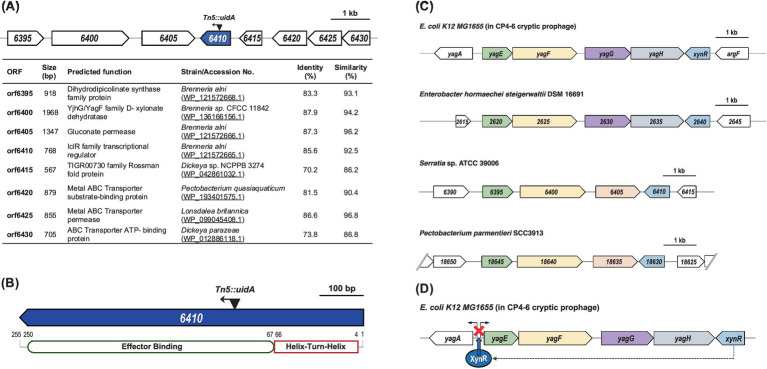
Organization of genes surrounding *orf6410* and comparison selected strains. **(A)** Locations of CS109 transposon insertion in *orf6410*, highlighted in blue, are shown by black triangles. The direction of the transposon insertion is indicated by the arrow heads. The predicted functions of genes surrounding *orf6410* based on BLASTx are indicated. **(B)** The predicted protein domains of Orf6410. The numbers above indicate the amino acid positions of each domain. **(C)** Comparison of the genomic region of *orf6410* with selected strains. Each ORF is represented by arrow blocks indication direction of transcription. Arrow blocks of the same color represent ORFs with similar predicted functions. **(D)** Proposed model for regulatory role of *E. coli xynR*. XynR binds the region the bidirectional transcriptional units *yagA* and *yagEF* repressing transcription of both (figure is adapted from Shimada et al. (2017)).

The transposon was inserted 296 bp from the start codon and in the same direction of transcription of *orf6410*, resulting in a *lacZ* transcriptional fusion (Figure 1A). Interrogation of the region surrounding *orf6410* showed that there are no predicted genes immediately upstream or downstream with which it could be co-transcribed. Since *orf6410* is predicted to be monocistronic and is divergently transcribed from the genes immediately downstream, the transposon insertion should not have any polar effects which had been confirmed by genetic complementation assays (Supplementary Figure 1). The *orf6410* gene product contains an *N*-terminal helix-turn-helix (HTH) and effector binding domains (Figure 1B) characteristic of IclR family transcriptional regulators (Molina-Henares et al., 2006). The transposon insertion is located within the effector binding domain of Orf6410. Based on the similarities, *orf6410* will now be designated as *iclR*.

A BLASTP search revealed that the amino acid sequence of Orf6410 had the highest degree of similarity and identity to IclR family transcriptional regulators from several species of *Brenneria* and *Pectobacterium* (Supplementary Table 2). Notably, the predicted protein was also similar to XynR transcriptional regulators from *Enterobacter hormaechei* (66.4% identity, 82.0% similarity) and *E. coli* (65.6% identity, 80.8% similarity). In *E. coli* K12, XynR (formerly YagI) is encoded on the CP4-6 cryptic prophage and has been shown to regulate the bidirectional transcription units: *yagA*, encoding an uncharacterised transcription factor and *yagEF*, encoding a xylonate dehydratase and a 2-keto-3-deoxygluconate aldolase, respectively (Shimada et al., 2017). Comparison of this genomic region in *E. coli* K12 to S39006, *E. hormaechei* and *P. parmentieri* (Figure 1C) revealed a similar gene organization across the strains. *E. coli xynR*, *yagE*, *yagF* and *yagG* (encoding a D-xylonate transporter) have homologous genes in all three strains analyzed but the putative S39006 *yagG* is located in a different genomic region. Homologs of *E. coli yagH* (encoding a xylosidase/arabinosidase) are found in *E. hormaechei* and *P. parmentieri* but not in S39006. Furthermore, it was predicted that S39006 *iclR* may play a role in the regulation of a two-gene operon composed of *orf6395* (encoding a putative dihydrodipicolinate synthase family protein) and *orf6400* (YjhG/YagF family D-xylonate dehydratase) similar to XynR’s predicted regulation of *yagEF* in *E. coli* K12 (Figure 1D) (Shimada et al., 2017).

### The *iclR* mutation attenuates GV production

**Figure 2:**
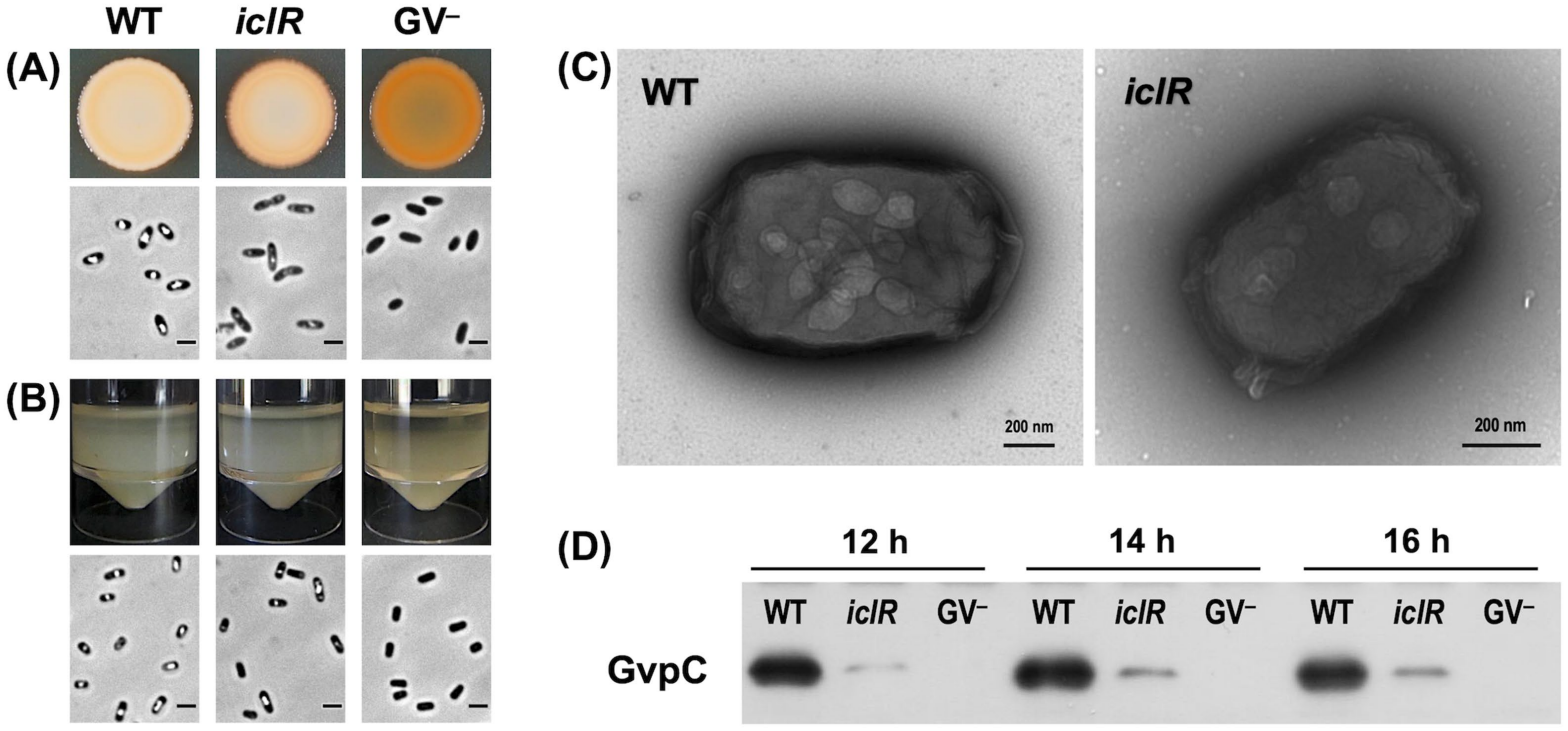
The disruption of *iclR* affects the GV phenotype of S39006. **(A)** Culture spots from a normalized number of cells (OD_600_ = 1.0) grown on LBA for 48 h at 30°C. **(B)** Images of the flotation assay taken after cultures were left static for 48 h at room temperature. PCM images of the cells from each culture immediately above. Scale bars on the PCM images represent 1 mm. **(C)** TEM images of negatively stained cells of S39006 wild type and *iclR* transposon mutant. **(D)** Immunodetection of gas vesicle structural protein GvpC in wild type S39006, *iclR* mutant and gas vesicle defective strain GPA1 at 12, 14 and 16 h of growth.

The *iclR* mutant was classified as an intermediate gas vesicle producer because the individual colonies appeared less opaque than the wild type on agar plates. Further, the culture spot of the transposon mutant appeared less opaque than the wild type but not translucent like the GV-defective GPA1 strain (Figure 2). The flotation ability of the mutants was also slightly decreased compared to the wild type, as seen in the static cultures where the air-liquid interface was more thinly populated by cells (Figure 2). PCM images of cells from culture spots and flotation assays further confirmed the gas vesicle phenotype of these mutants. Cultures of the intermediate gas vesicle producers were composed of a mixture of cells with and without gas vacuoles when viewed under PCM.

**Figure 3:**
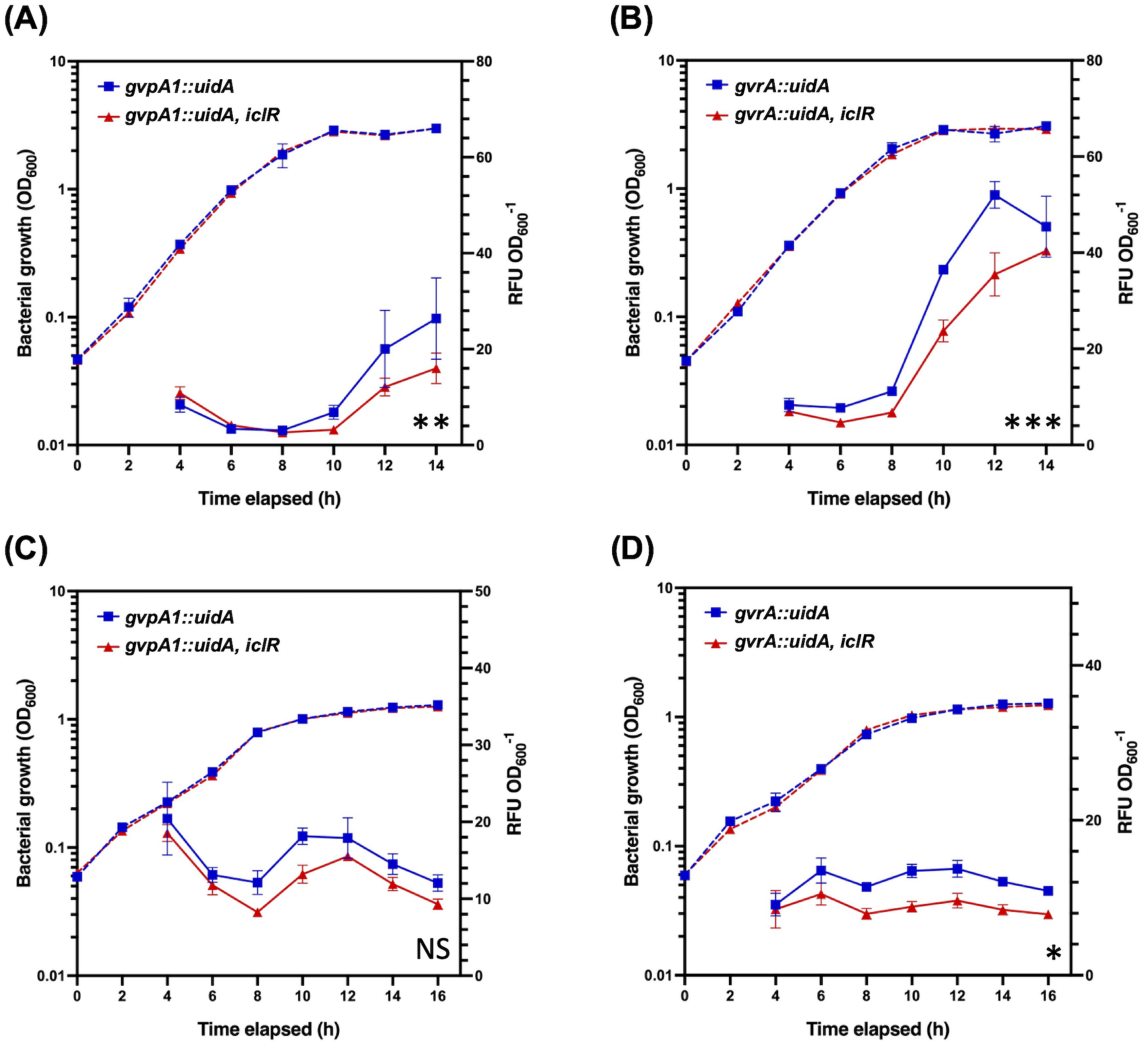
Expression of *gvpA1-gvpY* and *gvrA-gvrC* in wild type and the *iclR* mutant under aerobic and microaerophilic conditions. *β*-glucuronidase activity from *gvpA1::uidA* and *gvrA::uidA* chromosomal fusions was monitored over a growth curve in wild type (blue) and *iclR* mutant backgrounds (red) under **(A,B)** aerobic and **(C,D)** microaerophilic conditions. Solid lines indicate *β*-glucuronidase activity while dashed lines show OD_600_ measurements. Data shown are the mean values ± SD (*n* = 3) and asterisks indicate *p*-values comparing *β*-glucuronidase activity: *, *p* < 0.05; **, *p* < 0.01; ***, *p* < 0.001; NS, not significant.

The GV gene cluster of S39006 is 16.6 kb long and composed of 19 open reading frames (ORFs) responsible for gas vesicle morphogenesis arranged in two non-overlapping operons, *gvpA1-gvpY* and *gvrA*-*gvrC* (Ramsay et al., 2011). The impact of the disruption of *iclR* on the transcription of the GV cluster was also investigated using *gvpA1::uidA* and *gvrA::uidA* chromosomal fusions. The activity of *β*-glucuronidase (the product of the *uidA* gene) was used as a proxy for transcription of each operon and monitored over the course of a growth curve under aerobic or microaerophilic conditions. Under aerobic conditions, the expression of the *gvpA1-gvpY* operon in the *iclR* mutant was 2.12-fold, 1.66-fold, and 1.65-fold lower compared to the wild type at 10, 12, and 14 h of growth, respectively. Similarly, the expression of the *gvrA-gvrC* operon was 1.54-fold, 1.47-fold, and 1.12-fold lower at the same time points (Figures 3A,B). A similar trend was observed under microaerophilic conditions; although the difference in the *gvpA1-gvpY* transcription between the wild type and the transposon mutant was not statistically significant (Figure 3C).

### Disruption of IclR causes pleiotropic impacts on S39006 physiology

**Figure 4:**
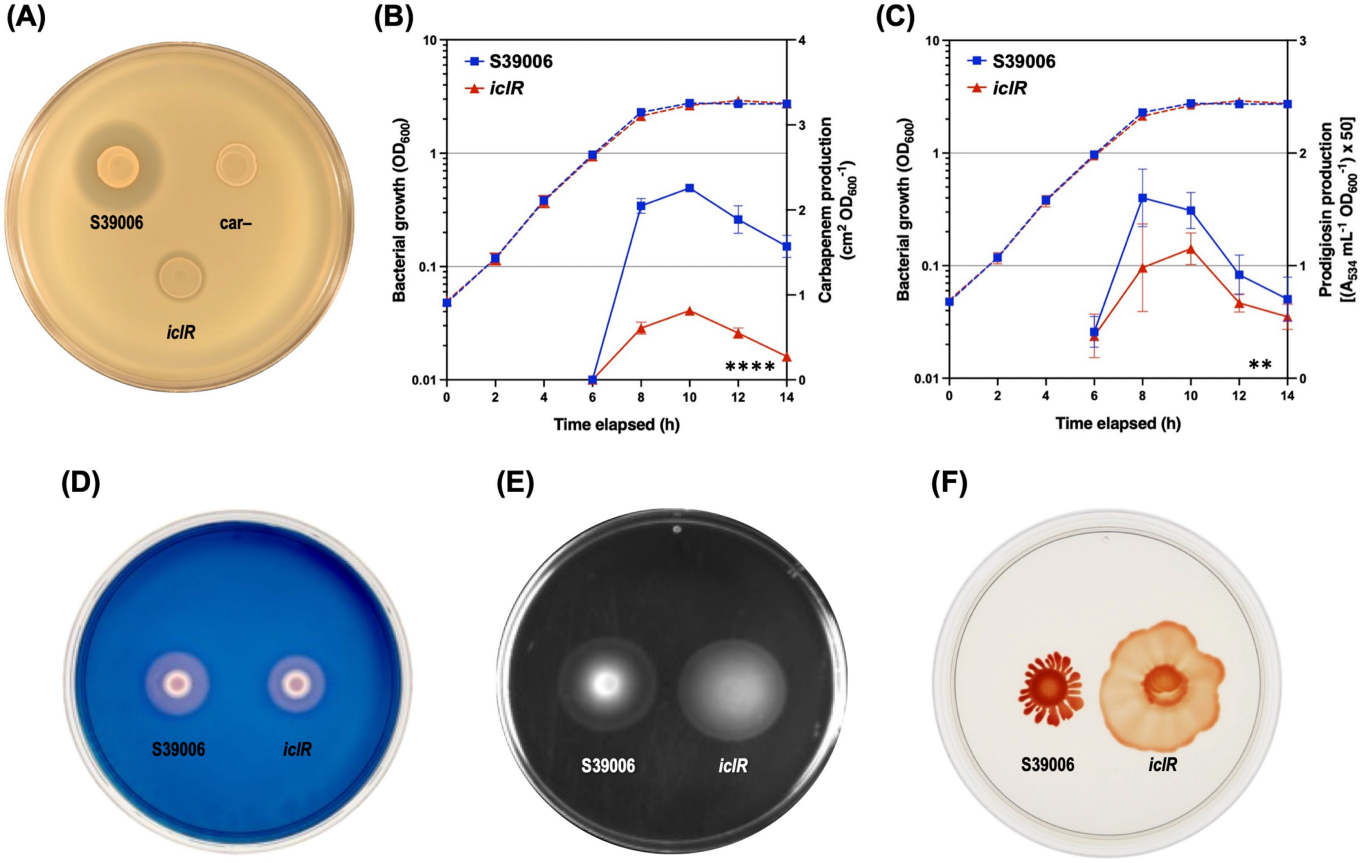
The *iclR* mutation is pleiotropic. Disruption of *iclR* decreased antibiotic production in S39006. **(A)** Carbapenem plate assay. The production of **(B)** carbapenem and **(C)** prodigiosin was also measured over the course of the growth curve for the S39006 wild type (blue) and the *iclR* transposon mutant (red). Dashed lines indicate OD_600_ readings while solid lines show carbapenem or prodigiosin production. Data shown are the mean values ± SD (*n* = 3) and asterisks indicate *p*-values comparing carbapenem or prodigiosin production: **, *p* < 0.01; ****, *p* < 0.0001. Plate assays for the production of **(D)** cellulase, as well as **(E)** swimming and **(F)** swarming motility also showed differences between the wild type and the mutant. For the plate assays, the cell densities of overnight cultures of the indicated strains were normalized prior to spotting onto the different agar plates. Images are representative of three biological replicates.

A series of phenotypic plate assays were performed with the *iclR* mutant to evaluate the pleiotropic effects of the mutation. Carbapenem production was greatly reduced in the mutant both in the plate spot tests and growth curve assay (Figure 4A). Results confirmed the significant reduction in carbapenem production in mutant with around a 2-fold decrease in the levels of antibiotic at the peak of its production (10 h) (Figure 4B). Production of prodigiosin and cellulase was also significantly reduced in the mutant (Figures 4C,D) while flagellar motility (swimming and swarming) was increased compared to the wild type (Figures 4E,F).

**Figure 5:**
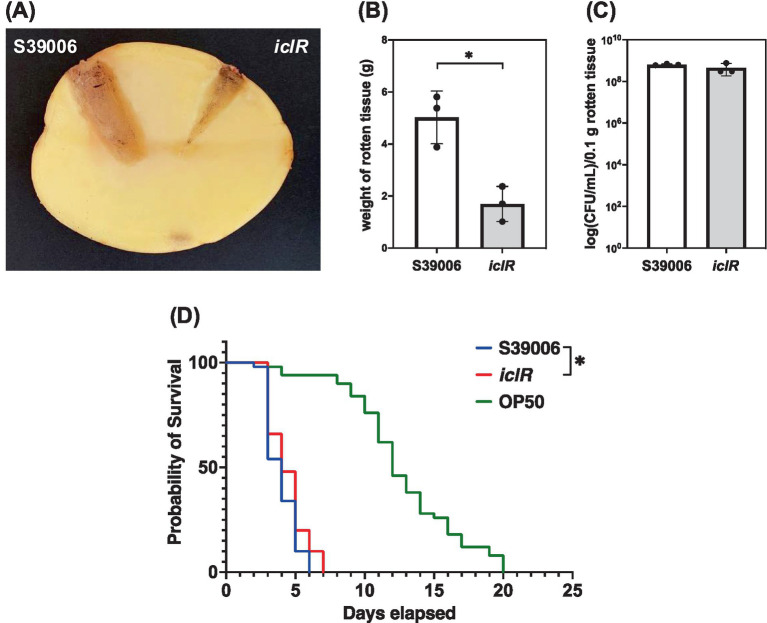
The *iclR* mutant showed attenuated phytopathogenicity and virulence in a *C. elegans* model. **(A)** Representative photo of a potato tuber comparing the areas of rotten tissue (stained with iodine for clarity) arising from the inoculation of S39006 wild type and the *iclR* transposon mutant. **(B)** Weight of rotten tissue generated and the **(C)** number of viable cells per 0.1 g rotten tissue were determined. **(D)** Survival curves for 50 stage L4 worms fed with S39006 (blue), the *iclR* mutant (red) and the control feeder strain *E. coli* OP50 (green). A log-rank test was performed to determine if there was a significant difference in survival between S39006 and the mutant and the asterisk indicates a *p*-value <0.05. Data shown are the mean values ± SD (*n* = 3) and the asterisk indicates *p*-values comparing the weight of rotten tissue: *, *p* < 0.05.

The impacts of the disruption of *iclR* on the virulence of S39006 *in planta* and in a *C. elegans* model were also established. Plant virulence of S39006 was assessed using potato rotting assays (Figure 5A). The mutant exhibited a significant reduction in virulence *in planta* with about a 3-fold decrease in the amount of rotten tissue after 5 days, compared to the wild type (Figure 5B). In addition, there was no significant difference in the viable cell counts recovered from the rotten tissues in both strains indicating that growth of the mutant is not impaired in potato tubers (Figure 5C). This reduction in plant virulence in the *iclR* mutant may be related to the decrease in its production of cellulase. Similarly, the mutant exhibited attenuated virulence against *C. elegans* with half the worms killed after 4 days and the entire population eliminated after 7 days. On average, worms fed with the mutant survived 1 day longer than with wild type which represents a slight, but statistically significant, reduction in virulence (Figure 5D).

### Differential proteomic analyses reveal potential regulatory roles of *iclR* in S39006

**Figure 6:**
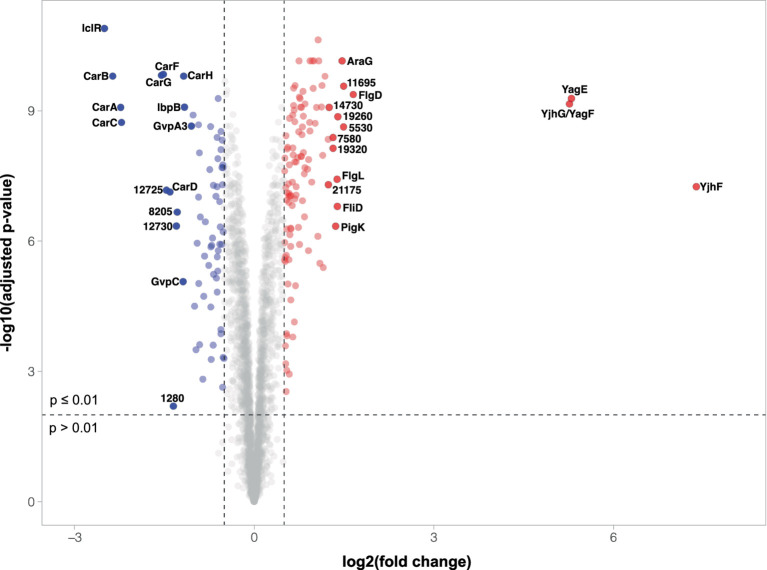
Volcano plot of differential protein abundances in the *iclR* mutant. The plot shows the relationship between fold change and adjusted *p*-values of the 2,959 proteins identified and quantified by TMT and LC–MS/MS. Downregulated proteins (*p* < 0.01 and log_2_FC < −0.5) are represented by blue dots while red dots indicated upregulated proteins (*p* < 0.01 and log_2_FC > 0.5). Gray dots represent proteins with no significant change in expression or changes of insufficient magnitude. The 15 most up-or downregulated proteins are highlighted and labeled with their protein name or corresponding ORF number where proteins are unnamed.

To determine any further impacts caused by the disruption of *iclR*, we performed a quantitative proteomic analysis comparing the intracellular proteomes of wild type S39006 and the transposon mutant. Moreover, information on proteins with altered abundances in the *iclR* mutant was used to identify potential mechanisms through which various phenotypes were affected in S39006. Analysis of the differentially expressed proteins focused on those with adjusted *p*-values of less than 0.01 and a log_2_FC cut-off of ±0.50. Using these parameters, 200 proteins in the *iclR* transposon mutant showed significantly altered abundances compared to the wild type. Of these, 81 proteins were downregulated (40.5%) while 119 proteins were upregulated (59.5%). Around 1,350 other proteins had a significant adjusted *p*-value (*p* < 0.01) but with log_2_FC values not within the cut-off. While such smaller changes in expression in some proteins (e.g., transcriptional regulators) may still have important physiological impacts, only those with highly altered abundances were analyzed for the purpose of this study. A volcano plot showing the protein abundances (log_2_FC) in the *iclR* mutant relative to the wild type as a function of statistical significance (−log of adjusted *p*-value) is presented in Figure 6.

Among the most downregulated proteins in the *iclR* mutant are those involved in gas vesicle and carbapenem biosynthesis, two key phenotypes that were attenuated in the mutant. Seventeen (17) of the 19 GV proteins were detected in the proteomic analysis, with log_2_FC ranging from −1.19 (GvpC) to −0.30 (GvrB) (Supplementary Figure 2A). The gas vesicle structural components GvpA1 (log_2_FC -0.97), GvpA3 (−1.05) and GvpC (−1.19) showed the most dramatic changes among the proteins encoded in the GV cluster. This is consistent with the decreased colony opacity and flotation ability observed in the *iclR* mutant. Furthermore, this also corroborated the observed reduction in transcription of the *gvpA1-gvpY* operon under aerobic conditions (Figure 3A). Expression of GV proteins from the *gvrA-gvrC* operon showed smaller, but still significant, changes ranging from −0.50 (GvpH) to −0.30 (GvrB). Unsurprisingly, the proteins involved in carbapenem synthesis (except for CarE which was not detected) and intrinsic resistance were all significantly less abundant in the mutant, with log_2_FC between −2.4 and − 1.2 (Supplementary Figure 2B). The reduced expression of the carbapenem biosynthetic enzymes CarA (−2.2), CarB (−2.4), CarC (−2.2) and CarD (−1.4) explains the impaired antibiotic activity of the *iclR* mutant. Moreover, the proteins responsible for providing intrinsic resistance to the carbapenem, CarF (−1.5) and CarG (−1.5), were also less abundant in the mutant. Despite this, the mutant was still resistant to exogenous carbapenem (Supplementary Figure 3) implying that low levels of CarF and CarG are still sufficient to confer carbapenem resistance in S39006. The abundances of the prodigiosin biosynthetic enzymes were also lower in the transposon mutant compared to the wild type (Supplementary Figure 2C) as expected. Of the 14 proteins encoded in the prodigiosin operon, 12 were detected in the proteomics analysis. Of these, 11 biosynthetic enzymes were downregulated (log_2_FC between −0.4 to −0.04) while one protein with unknown function was upregulated (PigK, log_2_FC = 1.36). Although the log_2_FC in the abundance of these enzymes are over the threshold of −0.5, they are still statistically significant except for PigA. The endoglucanase Orf13495 was also less abundant in the mutant by a log_2_FC of −0.53. This corroborates the observed reduction of cellulase production in the plate assay (Figure 4D) and may explain the attenuated phytopathogenicity of the *iclR* mutant demonstrated in the potato rotting assays (Figure 5A).

In addition to proteins that were expected to be downregulated in the *iclR* mutant based on its confirmed phenotypes – GV, carbapenem, prodigiosin and cellulase production, several other proteins were identified to be less abundant in the analysis. The 30 proteins with the most reduced abundance (*p* < 0.01) in the mutant, other than those involved in GV and carbapenem production, are summarized in Supplementary Table 3. Two uncharacterised proteins encoded by *orf12725* (−1.46) and *orf12730* (−1.30) were also heavily downregulated in the *iclR* mutant. Other notable proteins that were downregulated in the mutant are the heat shock proteins IbpA (−0.73) and IbpB (−1.16) and the dimethylsulfoxide reductase components DmsA (−0.95) and DmsB (−0.90).

On the contrary, the most abundant proteins in the *iclR* mutant are involved in chemotaxis and flagellar assembly (Supplementary Table 4) which supports the increased swimming and swarming motilities of the mutant observed in the plate assays (Figures 4E,F). The 30 most abundant proteins apart from chemotaxis or flagellar proteins are listed in Supplementary Table 5. Three proteins were extremely upregulated in the mutant – a dihydrodipicolinate synthase family protein YagE (Orf6395, log_2_FC 5.30), a D-xylonate dehydratase YagF (Orf6400, log_2_FC 5.26) and a gluconate permease YjhF (Orf6405, log_2_FC 7.38). As mentioned earlier, the two-gene *yagEF* operon is under the regulation of XynR in *E. coli* K12 (Figure 1D) (Shimada et al., 2017). A homologous operon is present in S39006 with a third gene, *yjhF*, located immediately downstream (Figure 1C). The significant increase in the abundance of YagE, YagF and YjhF in the *iclR* mutant provides strong evidence that *yagEF-yjhF* could be a single transcriptional unit under the repression of IclR.

Since IclR is a DNA-binding transcriptional regulator, it can also indirectly modulate expression of various genes via other regulators. The abundance of known regulators in the *iclR* mutant compared to the wild type are presented in Table 4. Several known regulators of gas vesicle and secondary metabolite production had significantly altered abundances including Rap (log_2_FC − 0.55), RpoS (−0.54), PstA (−0.37), PigX (−0.31), SmaR (−0.25), RbsR (−0.25), RsmA (−0.23), PhoB (−0.11), PigP (0.19), PigZ (0.32), PigT (0.32) and PigU (0.35). Although these regulators modulate either by activation or repression of expression, the overall effect in the *iclR* mutant was that GV and carbapenem production was diminished while prodigiosin production was slightly, but still significantly, reduced. Among the known activators of carbapenem production, only Rap was downregulated while PigP, PigZ and PigU were all upregulated. On the other hand, the repressors of carbapenem RpoS, SmaR, RsmA and PhoB were all less abundant in the mutant. Similarly, Rap was the only activator of prodigiosin synthesis that was less abundant while PigP, PigT and PigU were all upregulated. The repressors of prodigiosin synthesis RpoS, PstA, PigX and RsmA were upregulated and only PigZ was downregulated. Lastly, the GV activators Rap, RsmA and RbsR were both less abundant in the *iclR* mutant compared to the wild type which could have contributed to the decreased expression of the GV cluster. RpoS represses swimming motility, hence its downregulation could explain the increased swimming ability of the mutant. Consistently, the swimming motility activators PigZ, PigT and PigU were all upregulated. Swarming motility is known to be repressed by Rap, RpoS, PigS and RsmA which were all less abundant in the *iclR* mutant while the repressors PigT and RhlA were both upregulated. Several activators of cellulase production also showed altered abundances in the mutant. RpoS and RbsR were downregulated and PigP and PigZ were only slightly upregulated in the mutant.

**Table 4:** Known regulators with significantly altered abundances in the *iclR* mutant.

Name	log_2_FC	Adjusted *p*-value	Description	Effect on phenotype (+, activation; −, repression)						Reference
				GV	Car	Pig	Swim	Swarm	PCWDE	
Rap	-0.55	***	SlyA-like transcriptional regulator	+	+	+		−		Thomson et al. (1997) and Williamson et al. (2008) This study
RpoS	−0.54	***	RNA polymerase sigma factor	+	−	−	−	−	Cel + Pel –	Wilf and Salmond (2012), Wilf et al. (2011), and Quintero-Yanes et al. (2019)
PstA	−0.37	***	Phosphate transport system permease			−				Slater et al. (2003)
PigX	−0.31	***	GGDEF/EAL domain protein			−		−	Pel –	Fineran et al. (2007) and Williamson et al. (2008)
SmaR	−0.25	***	LuxR family transcriptional regulator	−	−	−		−		Thomson et al. (2000), Slater et al. (2003), Williamson et al. (2008), and Tashiro et al. (2016)
RbsR	−0.25	***	LacI family transcriptional regulator	+	+	+	−	−	Cel +	Lee et al. (2017)
RsmA	−0.23	***	RNA-binding protein	+	−	−		−		Ramsay et al. (2011), Wilf et al. (2011), and Williamson et al. (2008)
PhoB	−0.11	**	Phosphate regulon transcriptional regulatory protein (via activation of *rap* transcription)		−					Gristwood et al. (2009)
PhoR	−0.08	*	Phosphate regulon sensor protein (of PhoB)							Gristwood et al. (2009)
PigP	0.19	***	XRE family transcriptional regulator	+	+	+	−		Cel +	Fineran et al. (2005a) This study
PigZ	0.32	***	TetR family transcriptional repressor		+	−	+	−	+	Gristwood et al. (2008)
PigT	0.32	***	GntR family transcriptional regulator			+	+	+		Fineran et al. (2005b)
PigU	0.35	***	LysR transcriptional regulator		+	+	+			Fineran et al. (2005a) and Wilf et al. (2011)
RhlA	0.68	***	Alpha/beta hydrolase					+		Williamson et al. (2008)

*, *p* < 0.05; **, *p* < 0.01; ***, *p* < 0.001; GV, gas vesicles; Car, carbapenem; Pig, prodigiosin; Swim, swimming motility; Swarm, swarming motility; PCWDE, plant cell wall degrading enzymes; Cel, cellulase; Pel, pectate lyase; empty cells, not yet assessed or unchanged.

### IclR regulates transcription of the *yagE-yjhF* by binding its promoter

**Figure 7:**
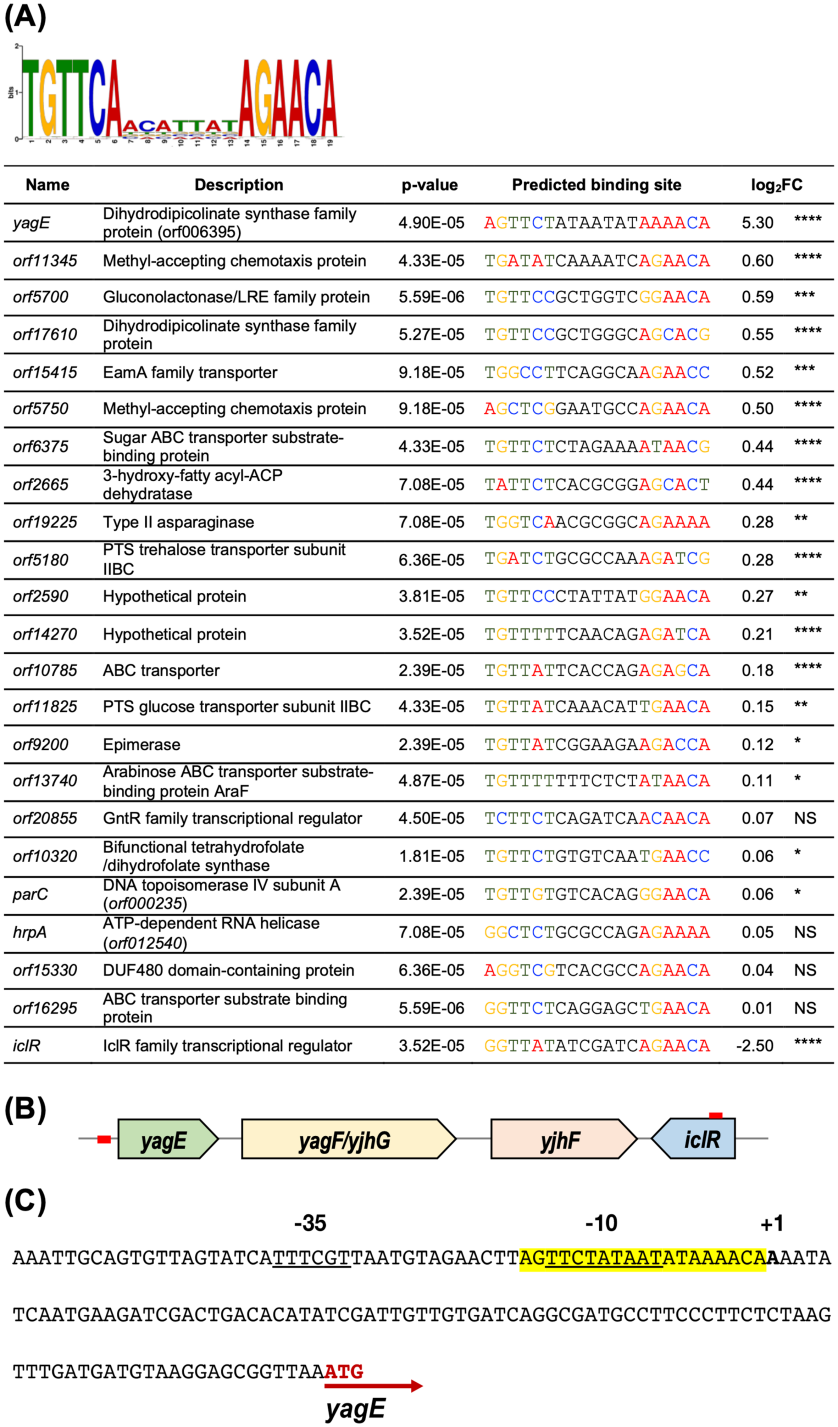
Predicted IclR binding sites in S39006. **(A)** The predicted binding sequence of *E. coli* XynR (Shimada et al., 2017) generated with MEME Suite and listed directly below are selected ORFs in the S39006 genome with predicted XynR binding sites immediately upstream. The log_2_FC of the proteins encoded by each gene is also indicated and the asterisks indicate *p*-values: *, *p* < 0.05, **, *p* < 0.01, ***, *p* < 0.001, ****, *p* < 0.0001. NS – not significant, *p* > 0.05. **(B)** Genomic organization of the *yagE-yjhF* operon and *xynR*. Red boxes upstream of *yagE* and within the *iclR* ORF indicate the predicted binding sites. **(C)** The putative promoter region of S39006 *yagE*. The predicted *yagE* transcriptional start site is denoted in boldface with a + 1 above. The potential-10 and -35 sites of its promoter are underlined and the predicted XynR box is highlighted in yellow. Finally, the translational start site of *yagE* is indicated with a red arrow.

**Figure 8:**
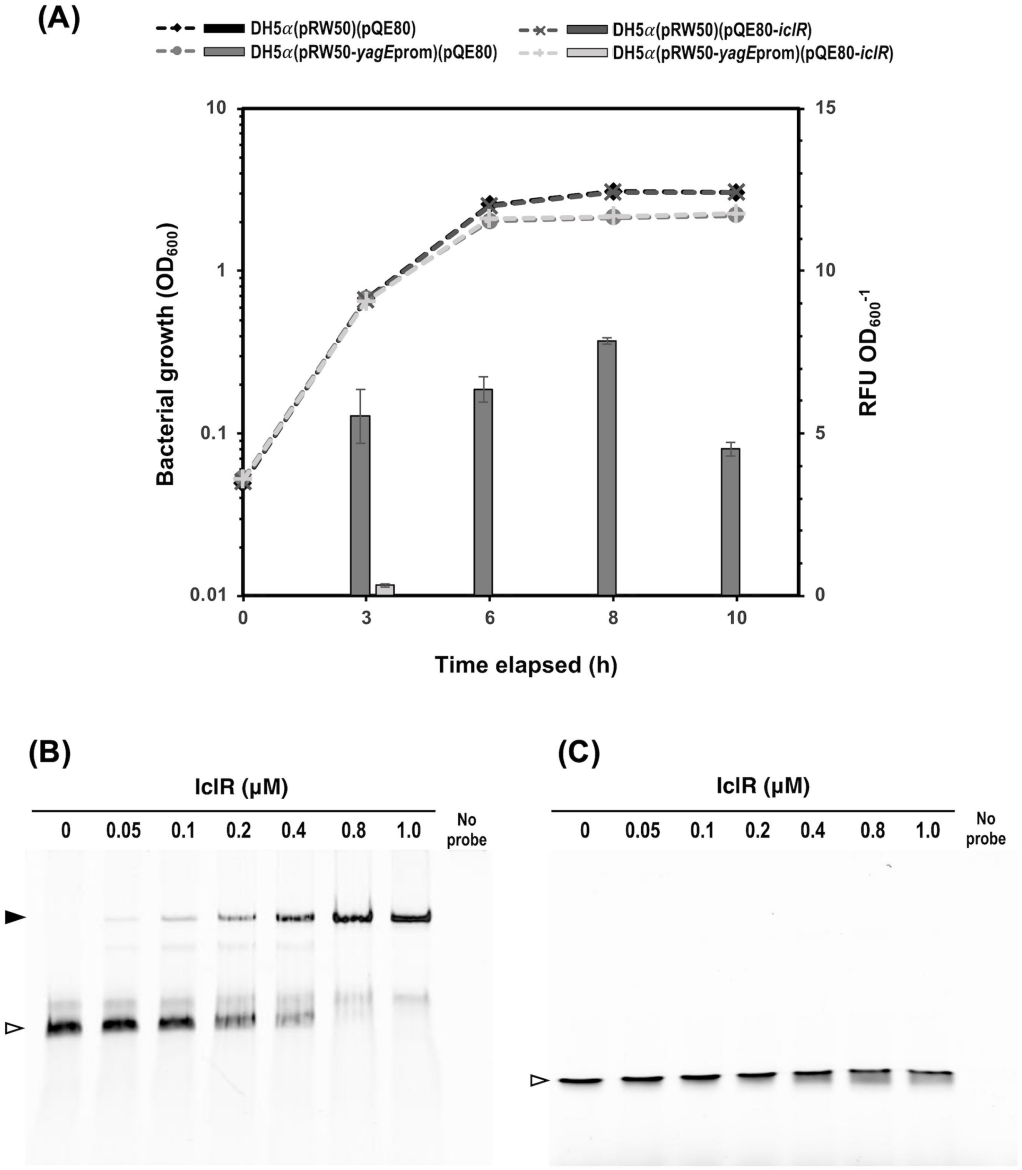
S39006 IclR directly regulates *yagE* by binding its promoter. **(A)** The *E. coli* DH5α cultures harboring pRW50 containing the putative *yagE* promoter fragment or pQE80 containing the *iclR* ORF were grown in flasks containing 25 mL of LB supplemented appropriate antibiotics and 0.1 mM IPTG to induce expression in pQE80. The activity of *β*-galactosidase protein, expressed from pRW50-*yagE*_prom_, dramatically reduced by co-expression of *iclR* from pQE80-*iclR*. The values are the average of three biological replicates ± standard deviation. Gel shift assays on Cy5-LUEGO-labeled **(B)** *yagE* and **(C)** *iclR* probes containing the predicted XynR box, titrated with the indicated concentrations of purified 6xHis-tagged IclR protein. Hollow arrowheads indicate the unbound probe while filled arrowheads indicate probes by the protein.

Differential proteomic analysis revealed that the cognate proteins of *yagE*, *yagF* and *yjhF* were extremely upregulated in the *iclR* transposon mutant. This strongly suggested that *yagEF-yhjF* locus could be an operon possibly under the control of IclR, similar to XynR in *E. coli* K12 (Shimada et al., 2017). The target site of XynR had been identified to be the palindromic sequence TGTTCAacattatAGAACA in *E. coli* K12 (Shimada et al., 2017). The Finding Individual Motif Occurrences (FIMO) program of the MEME Suite (Bailey et al., 2009; Grant et al., 2011) was used to scan for high-scoring motif occurrences of the consensus XynR box in the upstream sequences in the S39006 genome. Putative XynR binding sites were found in the upstream sequences of 76 genes (Supplementary Table 6). Some of the relevant putative XynR binding sequences are summarized in Figure 7A. Unsurprisingly, a XynR box was found upstream of *yagE* which supports the proposed role of XynR as a repressor of the putative *yagEF-yjhF* operon in S39006. A possible XynR binding site is also located upstream *orf17610*, a *yagE* homolog, which also encodes a dihydrodipicolinate synthase family protein (Figure 7B). Furthermore, a putative binding site was also found in the open reading frame of *iclR* which suggests that IclR might regulate its own synthesis (Figure 8A). Autoregulation is a typical feature of IclR transcriptional regulators in bacteria (Molina-Henares et al., 2006). XynR boxes were also found upstream of a wide variety of genes encoding methyl-accepting chemotaxis proteins (*orf5750* and *orf11345*), transporters (*orf5180*, *orf6375*, *orf10785*, *orf11825*, *orf13740*, *orf15415* and *orf16295*) and enzymes (*hrpA*, *parC*, *orf2665*, o*rf5700*, *orf9200*, *orf10320* and *orf19225*).

Further analysis of the sequence directly upstream of *yagE* confirmed the presence of promoter elements and a XynR box overlapping with the −10 site (Figure 7C). To investigate if IclR represses *yagEF-yjhF* transcription by binding the *yagE* promoter (*yagE*_prom_), a 150-bp fragment containing the *yagE*_prom_ was cloned into the *β*-gal transcriptional fusion vector pRW50, creating pRW50-*yagE*_prom_. DH5α cells were transformed with either pRW50 or pRW50-*yagE*_prom_ then the resulting strains were subsequently transformed with pQE80-*oriT* or pQE80-*iclR*. The constructed strains were grown in LB supplemented with the appropriate antibiotics and 0.1 mM IPTG and *β*-gal activity was monitored over the course of a growth curve (Figure 8A). Results showed that the *yagE*_prom_ is able to drive expression of the promoter-less *lacZ* gene as evidenced by the *β*-gal activity detected in the DH5α (pRW50-*yagE*_prom_) (pQE80-*oriT*). However, when IclR was expressed *in trans* in the DH5α (pRW50-*yagE*_prom_) (pQE80-*oriT*-*xynR*), the enzyme activity significantly dropped during exponential phase and was no longer detected throughout growth. This indicates that IclR could be directly repressing *yagE* transcription by binding to the XynR box within the promoter region.

Finally, to confirm that IclR binds the *yagE* promoter and investigate if it can bind its own ORF, gel shift assays were performed following the LUEGO method (Jullien and Herman, 2011). Cyanine-5 (Cy-5)-LUEGO-labeled *yagE* or *iclR* probes were mixed with increasing amounts of purified IclR and separated on a polyacrylamide gel. The mobility of the *yagE* probe on the gel was reduced and a complete band shift was achieved with 0.8 μM IclR (protein to DNA molar ratio of 32:1) (Figure 8B). This confirms that IclR represses transcription of the *yagEF-yjhF* operon by directly binding the *yagE* promoter. On the contrary, the mobility of the *iclR* probe was unchanged across the protein concentrations tested (Figure 8C) indicating that *iclR* transcription is unlikely to be negatively autoregulated.

## Discussion

The random transposon mutagenesis screen for gas vesicle mutants has led to the identification of another transcriptional regulator involved in carbohydrate metabolism, IclR, that affects multiple phenotypes in S39006. In *E. coli* K12, the IclR homolog XynR (formerly known as YagI) was first defined as a single-target transcriptional repressor of xylonate catabolism (Shimada et al., 2017). It controls catabolism of D-xylonate by altering transcription of the *yagEF* operon which encodes the catabolic enzymes 2-keto-3-deoxy gluconate aldolase and xylonate dehydratase, respectively (Shimada et al., 2017). At the time of this writing, this is the first report defining a XynR homolog as a pleiotropic regulator affecting multiple phenotypes in bacteria.

Using a combination of phenotypic, genetic and proteomic analyses, we have comprehensively characterized an *iclR* transposon mutant of S39006. The *iclR* mutant showed decreased flotation ability and produces fewer gas vesicles when compared to the wild type (Supplementary Figure 4). Reporter fusion assays further confirmed that expression of the *gvpA1-gvpY* operon was downregulated in the mutant but transcription of the *gvrA-gvrC* operon was only significantly reduced in the mutant under microaerophilic conditions. The disruption of *iclR* also had negative impacts on the production of secondary metabolites. Quantification of antibiotic activity over the course of a growth curve revealed a significant drop in carbapenem production (around a 3-fold decrease at peak production) in the mutant. Similarly, prodigiosin production was slightly reduced but was still statistically significant. The transposon mutant was also able to swim and swarm better than the wild type but its ability to kill *C. elegans* and cause soft rot in potato tubers were both reduced. The attenuation of the mutant’s plant pathogenicity could be in part explained by the decrease in its production of cellulase, a PCWDE.

A quantitative proteomics analysis comparing the intracellular proteins between the wild type S39006 and the *iclR* mutant corroborated the phenotypic changes in the mutant that were observed in assays. Furthermore, multiple pleiotropic regulators showed altered abundances in the mutant which suggests that IclR could be acting through control of other subordinate regulatory genes in complex regulatory networks.

All the GV proteins detected in the analysis were significantly downregulated. Notably, the decrease in abundances of the GV proteins expressed from the *gvrA-gvrC* operon were of lesser magnitude compared to those encoded in the *gvpA1-gvpY* operon. This is consistent with the results at the transcriptional level (using *β*-glu reporter assays) where only a slight decrease in expression of *gvrA-gvrC* was observed in the *iclR* mutant at 14 h (Figure 3B). Cell buoyancy in S39006 is positively regulated by low oxygen concentrations and this signal is likely to be transduced via the proteins encoded in *gvrA-gvrC* (Ramsay et al., 2011). Under microaerophilic conditions, only the transcription of the *gvrA-gvrC* operon was significantly lower in the *iclR* mutant background (Figure 3D). Therefore, this may suggest that IclR acts on *gvrA-gvrC* under suboptimal oxygen concentrations but not *gvpA1-gvpY*. It is possible that IclR indirectly regulates *gvrA-gvrC* expression via RbsR. RbsR has been proposed to bind upstream of *gvrA-gvrC* to regulate its transcription under both aerobic and microaerophilic conditions (Lee et al., 2017). RbsR was found to be less abundant in the *iclR* mutant and although this was determined under aerobic conditions, it is likely that this holds true under low oxygen concentrations.

The regulators Rap, RbsR, RsmA, SmaR and PigP which have previously been linked to gas vesicle formation showed altered abundances in the *iclR* mutant. This suggests that IclR could be acting indirectly through these regulators to control gas vesicle formation. The positive regulators of gas vesicle formation Rap, RbsR and RsmA were also all downregulated in the mutant which is consistent with the reduced gas vesicle phenotype. However, other known flotation regulators such as PigP was upregulated. The contrasting effects of these gas vesicle regulatory inputs could explain the intermediate or perturbed production of gas vesicle in the *iclR* mutant.

Several studies have linked carbon metabolism to prodigiosin production in *Serratia marcescens*. Contrasting reports have shown that cAMP-CRP can both positively or negatively impact pigment production (Kalivoda et al., 2010) although a more recent study suggested that the regulatory role of CRP is strain-dependent (Stella and Shanks, 2014). In a study on *S. marcescens* CMS376, cAMP-CRP has been shown to negatively regulate prodigiosin production via a homolog of S39006 PigP (Shanks et al., 2013). It is possible that catabolic regulators such as IclR, which senses “low quality” carbon sources, initiate cAMP-CRP-dependent repression of prodigiosin production.

Carbapenem production was dramatically reduced in the mutant but it remained resistant to the antibiotic despite the downregulation of the intrinsic resistance proteins (Supplementary Figure 3). This was also observed in the *floR* mutant (Quintero-Yanes et al., 2020) suggesting that low levels of CarF and CarG are sufficient to confer carbapenem resistance in S39006 to prevent suicide. Multiple previously defined regulators of carbapenem showed significantly altered abundances in the *iclR* mutant including Rap (log_2_FC -0.55), RpoS (−0.54), SmaR (−0.25), RbsR (−0.25), RsmA (−0.23), PhoB (−0.11), PigP (0.19), PigZ (0.32) and PigU (0.35). However, there is no obvious pattern to the changes in abundance of the positive regulators because Rap and RbsR were downregulated but PigP, PigZ and PigU were more abundant in the *iclR* mutant. In contrast, the negative regulators RpoS, SmaR, RsmA and PhoB were all downregulated for activation of carbapenem production. Given the dramatic reduction of carbapenem in mutant, it is likely that IclR indirectly activates antibiotic production via an unknown regulator(s).

Potato tuber assays demonstrated that the disruption of *iclR* greatly reduced phytopathogenicity of S39006 without affecting bacterial growth in plant tissue (Figure 5A). Production of PCWDEs and motility are shown to be important for virulence in many bacterial pathogens (Mulholland et al., 1993; Haiko and Westerlund-Wikström, 2013; Weller‐Stuart et al., 2017). However, the hyper motility of the *iclR* mutant did not result in increased plant pathogenicity. It is likely that while the *iclR* mutant cells are more motile, the reduced production of cellulase prevents effective degradation of the plant cell wall. Several studies have linked the role of carbohydrate metabolism to the virulence of plant pathogenic enterobacteria (Le Bouguénec and Schouler, 2011). In S39006, a mutation in *rbsR* attenuated virulence but also impaired growth in potato tubers (Lee et al., 2017). The *rbsR* mutant, like the *iclR* mutant, was hypermotile and showed a significant reduction in cellulase production. Potato rotting assays comparing the wild type S39006 and the *rbsR* mutant revealed a large reduction in the mutant’s ability to induce soft rot in the tuber (around a 40-fold difference in weight of rotten tissue recovered) (Lee et al., 2017). It has been suggested that RbsR could be co-regulating plant pathogenicity through known regulators or via new, unidentified, regulators (Lee et al., 2017). The abundance of defined virulence regulators detected in the *iclR* mutant, such as Rap and PigX, largely confirm their roles as virulence modulators in S39006. For instance, Rap (a positive regulator of plant pathogenicity) is downregulated in the mutant. PigX has been shown to attenuate plant pathogenicity by repressing the OpgG expression (involved in synthesis of osmoregulated periplasmic glucans), which is essential for full pathogenicity in S39006 (Fineran et al., 2007). PigX was slightly downregulated in the mutant but OpgG levels were unchanged. It is possible that the small change in abundance of PigX was not enough to elicit an upregulation of OpgG. Therefore, it is unlikely that IclR indirectly controls plant pathogenicity via PigX.

The *iclR* mutant showed slightly reduced ability to kill *C. elegans*, with worms surviving 1 day longer on average than in the wild type (Figure 5D). The disruption of *rpoS* in S39006 has been shown to upregulate flagellar motility and attenuate virulence in *C. elegans* but not *in planta* (Wilf and Salmond, 2012). Therefore, it is possible that IclR could be controlling virulence in *C. elegans* via RpoS. Biofilm formation has been also linked to the ability of bacteria to kill *C. elegans* (Joshua et al., 2003; Van Alst et al., 2007; Nandi et al., 2016) and could offer another explanation for attenuated virulence of the *iclR* mutant. A screen of the proteomics data for biofilm-associated proteins revealed that the positive regulator of biofilm formation YbaJ (an Hha homolog) was slightly less abundant in the mutant with a log_2_FC of −0.29, indicating possible impairment of biofilm formation.

Previous reports on S39006 have shown that mutants with disrupted genes linked to carbon metabolism (i.e., *rsmA*, *rbsR* and *floR*) exhibited an inverse relationship between flagellar motility and gas vesicle production (Lee et al., 2017; Quintero-Yanes et al., 2020; Ramsay et al., 2011). Therefore, it was unsurprising that the *iclR* mutant also showed increased swimming and swarming motilities. This phenotype was further corroborated by the upregulation of flagellar synthesis and chemotaxis proteins, including the flagellar transcriptional regulator FlhD (Supplementary Table 4). Moreover, multiple regulators linked to the control of flagellar motility showed altered abundances in the *iclR* mutant. The abundance of each regulator was mostly consistent with the expected effect in cell motility. Notably, IclR could be acting indirectly through Rap, RpoS and RhlA (Thomson et al., 1997; Wilf and Salmond, 2012; Williamson et al., 2008) to upregulate flagellar motility and diminish gas vesicle production.

In *Serratia* and *E. coli*, glucose represses flagellar motility by downregulating the expression of adenylate cyclase, CyaA, which is involved in cAMP synthesis (Adler and Templeton, 1967; Silverman and Simon, 1974; Stella et al., 2008). Moreover, conditions that decrease cAMP levels such as a high glucose concentrations have been shown to inhibit flagellar synthesis via CRP-dependent repression of *flhDC* (Soutourina et al., 1999; Liu et al., 2005; Stella et al., 2008). Furthermore, it has been shown in *E. coli* that cell motility is upregulated as the quality of carbon source decreases (Liu et al., 2005). In liquid environments, it is sensible for S39006 to inhibit flagellar synthesis and remain afloat when the availability or quality of carbon sources are favorable. This further suggests that S39006 could be using xylonate catabolism (via IclR) to sense less-preferred carbon sources, such as D-xylonate, in the environment to activate flagellar motility. This study has also confirmed that IclR represses xylonate catabolism in S39006 by binding the *yagEF-yjhG* promoter, similar to its homolog XynR in *E. coli* K12. XynR binding in *E. coli* K12 is inhibited by D-xylonate (Shimada et al., 2017) but we were unable to confirm this in S39006. However, if D-xylonate also inhibits IclR binding in S39006 then the following regulatory model is proposed. The presence of D-xylonate activates transcription of its catabolic genes, signaling the switch from flotation to flagellar motility to facilitate migration to more favorable environments.

Genetic and proteomic analysis of the *iclR* mutant suggested several potential mechanisms through which this novel regulator acts to control multiple phenotypes in S39006, directly or indirectly, via subordinate genes. We also presented a new way in which S39006 modulates gas vesicle biogenesis in response to environmental inputs such as xylonate, mediated by IclR. Given its broad regulatory role and involvement in xylonate-associated pathways in S39006, we designate this IclR-family transcriptional regulator XyrR (*Xy*lonate *r*esponse *R*egulator).

Finally, although this work has contributed novel and substantial information on how S39006 key phenotypes are modulated in response to biotic and abiotic factors, it has further highlighted the extreme complexity of the regulatory network governing these phenotypes. Future work should focus on deciphering the extent of such an intricate network using a multi-omics approach combined with systems modeling to develop a comprehensive understanding of the physiology of this enterobacterium. These experiments should be carried out in tightly controlled physiological conditions in batch cultures and in chemostats under rigorous environmental manipulations.

## Data Availability

The original contributions presented in the study are included in the article or supplementary material, further inquiries can be directed to the corresponding author.
