# Supplementary material for: The IclR-family transcriptional regulator XyrR controls flotation, motility, antibiotic production and virulence in Serratia sp. ATCC 39006
Source: Front Microbiol. 2025 Jan 15;15:1500889. doi: 10.3389/fmicb.2024.1500889 (PMC11778176; doi:10.3389/fmicb.2024.1500889)
Supplement: Supplementary file 1 [file Data_Sheet_1.pdf]

Supplementary Material

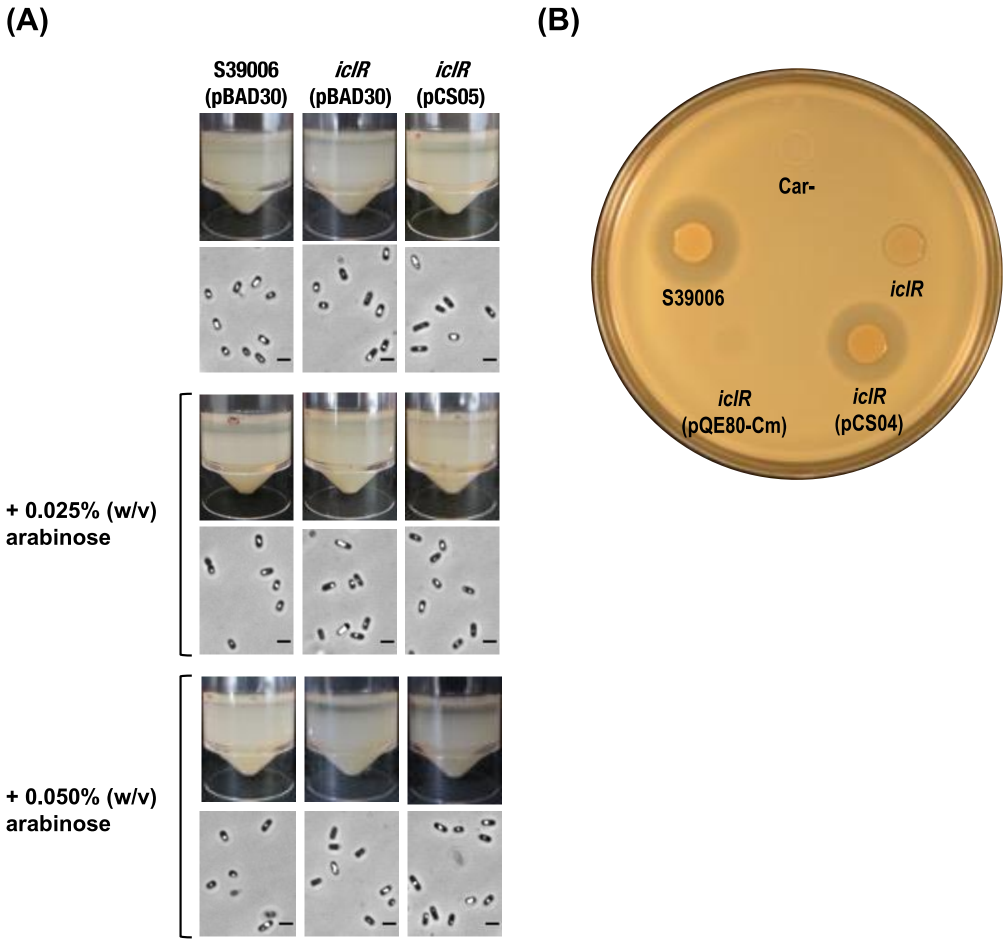

Supplementary Figure 1. The expression of *iclR in trans* complemented carbapenem production but not flotation. (A) Effect of ectopic expression of *iclR* from pCS05 induced with the indicated arabinose concentrations on flotation ability of the mutant. The GV phenotype of the other gas vesicle mutants had been complemented using the same expression system. However, proteomics data later revealed that the mutant has impaired arabinose transport which could have resulted in modulation of GV expression that is independent of the *iclR* mutation. Therefore, it is possible that the *iclR* mutation had an impact on arabinose metabolism of CS109. **(B)** Basal expression of *iclR* from pCS04 restored carbapenem production in the mutant.

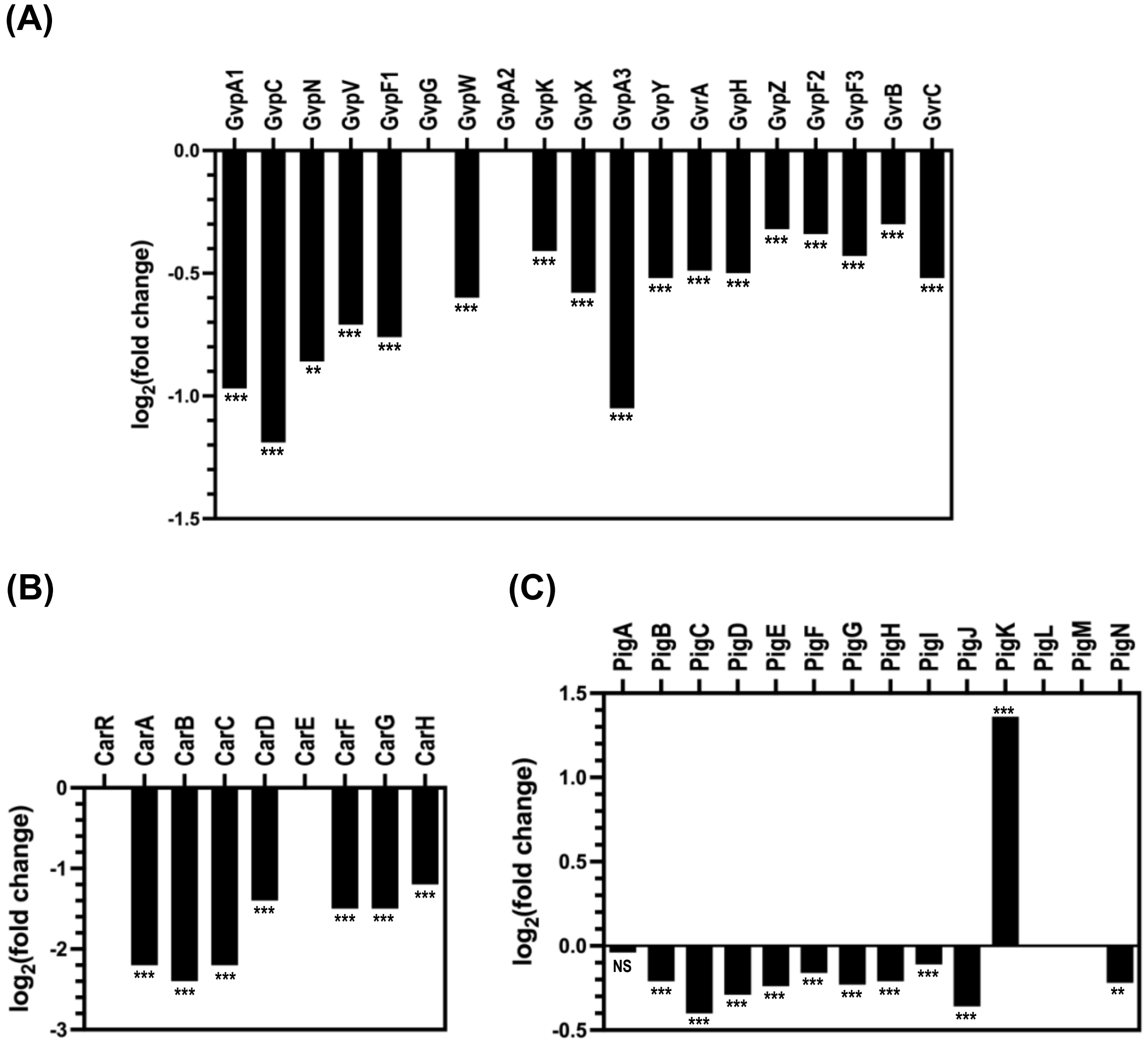

Supplementary Figure 2. IclR downregulates expression of gas vesicle (GV), carbapenem (Car) and prodigiosin (Pig) proteins. Bars indicate the difference of abundance of **(A)** GV, **(B)** Car, and **(C)** Pig proteins in the *iclR* mutant compared to S39006. Asterisks represent adjusted p-values: **, $p < 0.01$; ***, $p < 0.001$. NS indicate no statistical significance. Proteins which were not detected in the analysis are represented by spaces without bars.

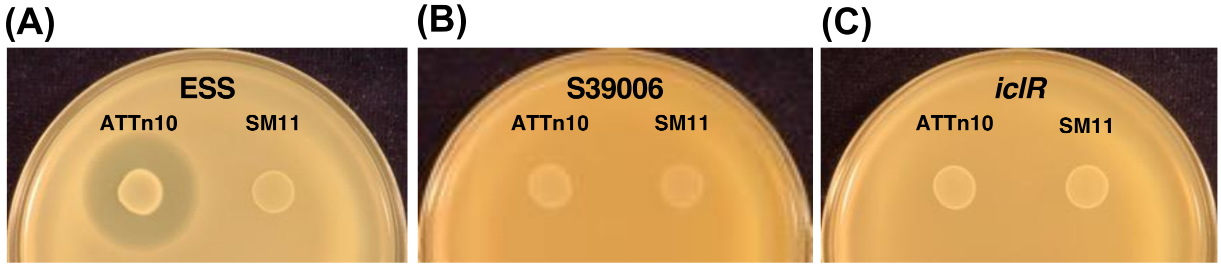

Supplementary Figure 3. The *iclR* transposon mutant is still resistant to carbapenem. Normalised cultures of the carbapenem producing strain *P. carotovorum* ATTn10 and the carbapenem mutant *P. carotovorum* SM11 were spotted on top lawns of **(A)** the β-lactam supersensitive strain *E. coli* ESS, **(B)** S39006 wild type and **(C)** the *iclR* mutant CS109. All images are representative of three biological replicates.

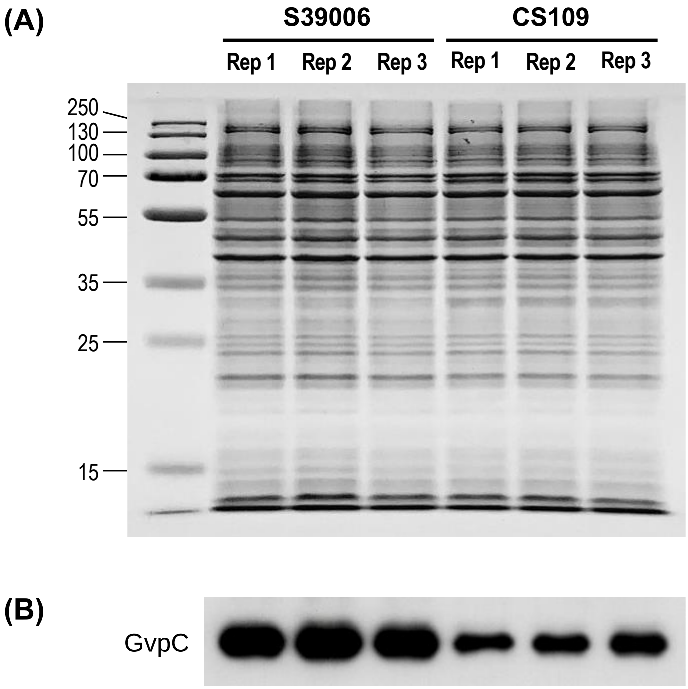

Supplementary Figure 4. Western blot detecting GvpC in the wild-type and *iclR* mutant. Intracellular proteins were extracted from14-hour cultures normalized to an OD_{600} of 1.0. **(A)** SDS-PAGE profiles of the intracellular protein samples for each of the three replicates of S39006 and CS109. The first lane contains shows protein ladder (Thermo Scientific™ PageRuler™ Plus) with the size of each band in kDa indicated. **(B)** Western blot detecting GvpC in each of the three replicates.

Supplementary Table 1. Composition of buffers used in Gibson assembly.

Buffers	Components
5X ISO Buffer<br>(per 6 mL)	(3 mL 1M Tris-HCl [pH 7.5])<br>(150 μL 2M $MgCl_2$)<br>240 μL 100mM dNTPs<br>300 μL 1M DTT<br>300 μL 100mM NAD<br>1.5 g PEG-8000
Gibson reaction buffer<br>(per 1.2 mL)	320 μL 5X ISO buffer<br>0.64 μL T5 exonuclease<br>20 μL Phusion DNA polymerase (2 U/μL)<br>160 μL Taq DNA ligase (40 U/μL)

Supplementary Table 2. Similarity search of the predicted amino acid sequence of *orf6410* using BLASTP

Species	Protein	Length (aa)	Identity (%)	Similarity (%)	Accession No.
Brenneria alni	IclR	252	85.6	92.5	WP_121572665.1
Brenneria sp. hezel4-2-4	IclR	252	85.2	92.2	WP_172291118.1
Brenneria sp. L3-3C-1	IclR	252	84.8	91.8	WP_199378489.1
Brenneria sp. CFCC 11842	IclR	252	84.0	90.1	WP_136166158.1
Pectobacterium wasabiae	IclR	252	83.6	93.3	WP_005975072.1
Pectobacterium parmentieri	IclR	252	83.2	92.9	WP_103807983.1
Enterobacter hormaechei	XynR	253	66.4	82.0	WP_063150734.1
Escherichia coli	XynR	252	65.6	80.8	EFG2864720.1

Supplementary Table 3. Other downregulated proteins in the *iclR* mutant ($p < 0.01$).*

Protein ID	Name	Description	log_2FC	Adjusted p-value
**A0A2I5T4I6**	IclR	IclR family transcriptional regulator (Orf6410)	-2.50	1.27E-11
**A0A2I5TQI9**	Orf12725	Uncharacterised protein	-1.46	6.75E-08
**A0A2I5TK25**	Orf12730	Uncharacterised protein	-1.30	4.51E-07
**A0A2I5THS7**	Orf8205	Uncharacterised protein	-1.28	2.16E-07
**A0A2I5T2T5**	IbpB	Heat shock chaperone (Orf3065)	-1.16	8.26E-10
**A0A2I5T5G4**	Orf8225	DUF4336 domain-containing protein	-1.02	1.26E-09
**A0A2I5TME6**	HyfH	Hydrogenase 4 subunit H (HycF, Orf17295)	-0.99	3.16E-05
**A0A2I5T997**	DmsA	Dimethylsulfoxide reductase subunit A (Orf15805)	-0.95	1.12E-06
**A0A2I5TMD4**	HycH	Formate hydrogenlyase maturation protein (HyfJ, Orf17305)	-0.93	9.72E-08
**A0A2I5TC94**	Orf21805	Lactaldehyde reductase	-0.93	2.10E-09
**A0A2I5T7N3**	NarI	Respiratory nitrate reductase subunit gamma (Orf12625)	-0.93	9.46E-06
**A0A2I5T987**	Orf15675	Uncharacterised protein	-0.91	9.32E-09
**A0A2I5TME0**	HyfI	NADH-quinone oxidoreductase subunit (HycG/NuoB, Orf17300)	-0.91	0.0002
**A0A2I5T9B3**	DmsB	Dimethylsulfoxide reductase chain B (Orf15800)	-0.90	2.80E-07
**A0A2I5THT4**	Orf8210	Radical SAM protein	-0.84	1.87E-05
**A0A2I5TL38**	PepT	Peptidase T (Orf14760)	-0.83	2.22E-06
**A0A2I5TPU6**	Orf22230	ATP-independent periplasmic protein-refolding chaperone	-0.81	3.61E-07
**A0A2I5TLS8**	Orf16075	Uncharacterised protein	-0.74	2.26E-08
**A0A2I5T2U4**	IbpA	Small heat shock protein (Orf3060)	-0.73	2.31E-09
**A0A2I5T195**	Orf20	Xylanase	-0.72	3.29E-05
**A0A2I5T9W0**	Orf17020	Uncharacterised protein	-0.72	1.37E-06
**A0A2I5TM90**	SpeF	Ornithine decarboxylase (Orf16980)	-0.72	0.0005
**A0A2I5TPY4**	AsrC	Sulfite reductase subunit C (Orf22460)	-0.70	8.54E-07
**A0A2I5TJI5**	BssS	Biofilm transcriptional regulator (Orf11730)	-0.68	0.0002
**A0A2I5TDJ9**	AlsS	Acetolactate synthase (Orf21810)	-0.68	5.20E-08
**A0A2I5T8V4**	Orf15000	Uncharacterised protein	-0.68	5.84E-06
**A0A2I5TLH2**	Orf15410	Amidinotransferase	-0.64	9.32E-08
**A0A2I5TKT8**	Orf14170	KilA domain-containing DNA-binding protein	-0.63	7.12E-06
**A0A2I5T397**	Orf3905	Class I SAM-dependent methyltransferase	-0.62	5.60E-08
**A0A2I5T9L7**	Orf16490	Heat-shock protein	-0.62	4.12E-09

*excluding GV and carbapenem proteins presented in Supplementary Figure 2 (A and B)

Supplementary Table 4. Abundance of proteins involved in chemotaxis and flagellar biosynthesis and assembly in the *iclR* mutant ($p < 0.01$).

Protein ID	Name	Description	log_2FC	Adjusted p-value
**A0A2I5T731**	FlgD	Basal-body rod modification protein (Orf011445)	1.65	4.23E-10
**A0A2I5TJG3**	FliD	Flagellar hook-associated protein 2 (Orf011570)	1.39	1.59E-07
**A0A2I5T773**	FlgL	Flagellar hook-filament junction protein (Orf11485)	1.38	3.78E-08
**A0A2I5TNH2**	Orf19320	Methyl-accepting chemotaxis protein	1.32	7.33E-09
**A0A2I5TL40**	Orf14730	Methyl-accepting chemotaxis protein	1.25	8.36E-10
**A0A2I5TQV1**	Orf21175	Chemotaxis protein	1.24	5.04E-08
**A0A2I5T713**	FlgK	Flagellar hook-associated protein 1 (Orf11480)	1.24	4.56E-09
**A0A2I5T4U2**	Orf7015	Methyl-accepting chemotaxis protein	1.14	1.06E-08
**A0A2I5T734**	FliH	Flagellar assembly protein (Orf11540)	1.12	8.18E-10
**A0A2I5TJF3**	FliG	Flagellar motor switch protein (Orf11545)	1.08	7.13E-11
**A0A2I5TJI1**	FliC	Flagellin (Orf11575)	1.04	8.36E-10
**A0A2I5TCN4**	Orf22655	Methyl-accepting chemotaxis protein	0.96	4.39E-08
**A0A2I5TJE0**	FlgI	Flagellar P-ring protein (Orf11470)	0.93	1.86E-09
**A0A2I5T2S7**	Orf2960	Methyl-accepting chemotaxis protein	0.91	1.33E-08
**A0A2I5T704**	MotA	Flagellar motor stator protein (Orf11360)	0.84	2.77E-07
**A0A2I5TMV9**	Orf18215	Methyl-accepting chemotaxis protein	0.84	2.84E-08
**A0A2I5T728**	CheB	Chemotaxis response regulator (Orf11390)	0.82	1.33E-09
**A0A2I5T787**	FliI	Flagellum-specific ATP synthase (Orf11535)	0.81	2.35E-09
**A0A2I5T4L4**	Orf6560	Methyl-accepting chemotaxis protein	0.79	1.12E-08
**A0A2I5T6Z9**	MotB	Motility protein (Orf11365)	0.79	1.13E-08
**A0A2I5TJD2**	CheY	Chemotaxis protein (Orf11395)	0.78	8.26E-10
**A0A2I5T707**	Orf11385	Chemotaxis protein methyltransferase	0.75	7.13E-11
**A0A2I5TQH5**	FlgH	Flagellar L-ring protein (Orf11465)	0.74	3.06E-09
**A0A2I5T9J2**	Orf16050	Methyl-accepting chemotaxis protein	0.74	4.84E-07
**A0A2I5T763**	FlgA	Flagella basal body P-ring formation protein (Orf11430)	0.70	5.60E-08
**A0A2I5TJD3**	FlhD	Flagellar transcriptional regulator (Orf11350)	0.69	1.07E-05
**A0A2I5TJF1**	FliT	Flagella biosynthesis regulatory protein (Orf11560)	0.68	1.79E-09
**A0A2I5TB03**	Orf19165	Methyl-accepting chemotaxis protein	0.65	0.0002
**C5J9G8**	FlhA	Flagellar biosynthesis protein (Orf11410)	0.62	9.04E-08
**A0A2I5T746**	FlgE	Flagellar hook protein (Orf11450)	0.61	1.33E-06
**A0A2I5T6Z1**	CheA	Chemotaxis protein (Orf11370)	0.60	9.04E-08
**A0A2I5TJC4**	Orf11345	Methyl-accepting chemotaxis protein	0.60	7.11E-07
**A0A2I5TJE5**	CheW	Chemotaxis protein (Orf11375)	0.59	1.75E-08
**A0A2I5T727**	FliL	Flagellar protein (Orf11520)	0.51	1.08E-06
**A0A2I5T2Y6**	Orf3330	Methyl-accepting chemotaxis protein	0.51	2.76E-06
**A0A2I5T4A0**	Orf5750	Methyl-accepting chemotaxis protein	0.50	6.59E-07
**A0A2I5T716**	FlhB	Flagellar biosynthetic protein (Orf11405)	0.47	3.16E-07
**A0A2I5T7L5**	Orf12520	Chemotaxis protein	0.43	2.25E-05
**A0A2I5T700**	FlgN	Flagellar biosynthesis protein (Orf11420)	0.37	4.19E-07
**A0A2I5TEC6**	Orf1395	Methyl-accepting chemotaxis protein	0.36	0.0001
**A0A2I5TJF8**	FliM	Flagellar motor switch protein (Orf11515)	0.36	1.08E-08
**A0A2I5T4V5**	Orf6985	Methyl-accepting chemotaxis protein	0.29	8.78E-05

Supplementary Table 5. Other upregulated proteins in the *iclR* mutant ($p < 0.01$).*

Protein ID	Name	Description	log_2FC	Adjusted p-value
**A0A2I5T4M1**	YjhF	Gluconate permease (Orf6405)	7.38	5.60E-08
**A0A2I5T4I0**	YagE	Dihydrodipicolinate synthase family protein (Orf6395)	5.30	5.22E-10
**A0A2I5T4I1**	YagF	YjhG/YagF family D-xylonate dehydratase (Orf6400)	5.26	6.98E-10
**A0A2I5T757**	Orf11695	Uncharacterised protein	1.49	2.72E-10
**A0A2I5TGF3**	Orf5530	Uncharacterised protein	1.49	2.35E-09
**A0A2I5T8C9**	AraG	L-arabinose import ATP-binding protein (Orf13735)	1.47	7.13E-11
**A0A2I5TB24**	Orf19260	Uncharacterised protein	1.40	1.37E-09
**A0A2I5TBR1**	PigK	RedY (Orf20645)	1.36	4.55E-07
**A0A2I5TD14**	Orf7580	Amino acid ABC transporter substrate-binding protein	1.32	4.13E-09
**A0A2I5TKU9**	Orf14265	Uncharacterised protein	1.18	1.61E-10
**A0A2I5T864**	Orf13630	DNA replication terminus site-binding protein	1.15	4.08E-06
**A0A2I5TDQ4**	Orf220	LysR family transcriptional regulator	1.13	2.63E-10
**A0A2I5TH12**	Orf6780	Alkyl hydroperoxide reductase subunit F	1.10	3.24E-06
**A0A2I5T744**	Orf11590	DegT/DnrJ/EryC1/StrS family aminotransferase	1.07	2.34E-11
**A0A2I5TC92**	CspA	RNA chaperone/antiterminator (Orf21685)	1.06	7.77E-07
**A0A2I5TAV3**	ComEA	Competence protein (Orf18930)	1.02	1.94E-09
**A0A2I5TKQ6**	Orf14040	DUF1315 domain-containing protein	1.00	7.13E-11
**A0A2I5TKD3**	Orf13145	NAD(P)-dependent oxidoreductase	0.99	3.05E-10
**A0A2I5TJL5**	Orf11585	Glyco_trans_2-like domain-containing protein	0.97	7.13E-11
**A0A2I5T761**	Orf11600	Carbamoyl-phosphate synthase small subunit	0.93	7.13E-11
**A0A2I5T9K3**	Orf16385	Uncharacterised protein	0.91	1.66E-06
**A0A2I5TJG2**	Orf11595	Class I SAM-dependent methyltransferase	0.89	2.21E-08
**V3TWJ7**	Orf15135	DNA polymerase III subunit theta	0.85	6.95E-10
**A0A2I5TPR1**	LsrD	Autoinducer 2 ABC transporter permease (Orf21945)	0.85	2.04E-08
**A0A2I5TJ18**	Orf10855	Non-ribosomal peptide synthetase	0.82	9.14E-09
**A0A2I5T777**	Orf11605	Gfo/Idh/MocA family oxidoreductase	0.82	3.58E-10
**A0A2I5TDP9**	Orf205	FAA hydrolase family protein	0.81	1.18E-07
**A0A2I5TM77**	ArgF	Ornithine carbamoyltransferase (Orf16995)	0.80	3.57E-09
**A0A2I5T820**	Orf13480	Uncharacterised protein	0.79	8.36E-10
**A0A2I5TJI3**	Orf11580	Glyco_trans_2-like domain-containing protein	0.77	5.22E-10

*excluding flagellar motility and chemotaxis proteins

Supplementary Table 6. Genes with putative XynR binding sites immediately upstream of ORF predicted by FIMO.

ORF	Description	p-value	Matched Sequence
235	DNA topoisomerase IV subunit A	2.39E-05	TGTTGTGTCACAGGGAACA
575	Shikimate kinase	9.18E-05	AGTTCTTCGACTCCGATCA
1105	Fic family protein	6.36E-05	TGACCTGGCAAGCAGAGCA
1370	o-acetyltransferase	1.81E-05	TGTTCTTATCCACAGTACC
2305	Thiamine phosphate syntase	7.08E-05	TTCCCTCCACCGCAGAACA
2590	Hypothetical protein	3.81E-05	TGTTCCCTATTATGGAACA
2665	3-hydroxy-fatty acyl-ACP dehydratase	7.08E-05	TATTCTCACGCGGAGCACT
2895	Sigma-70 family RNA polymerase sigma factor	4.33E-05	TGTTCTATATTCGCCAACA
3000	LacI family transcriptional regulator	4.96E-05	TGTTATAAAAAATACAACA
4045	Phosphogluconate dehydratase	1.31E-05	TGTTCCGCCATGCAGAAAA
4180	Pirin family protein	4.53E-05	TGTTCGTATTTTTTGAACA
4615	PLP-dependent aminotransferase family protein	5.27E-05	TGAACTGGCTGGCAGAGCA
5180	PTS trehalose transporter subunit IIBC	6.36E-05	TGATCTGCGCCAAAGATCG
5620	Glucose-6-phosphate isomerase	2.46E-05	TGTTGTGCAAGCCAGAAGA
5675	Transketolase	1.81E-05	TGTCCTCTCGTAAAGAACT
5700	Gluconolactonase	5.59E-06	TGTTCCGCTGGTCGGAACA
5750	Methyl-accepting chemotaxis protein	9.18E-05	AGCTCGGAATGCCAGAACA
5910	Carbamoyl-phosphate synthase small subunit	9.18E-05	GGTTCTGGAAGACGGAACC
6370	Alpha mannosidase	4.50E-05	TGTTTTTTACCGAAGAAAA
6375	Sugar ABC transporter substrate-binding protein	4.33E-05	TGTTCTCTAGAAAATAACG
6395	Dihydrodipicolinate synthase family protein	4.90E-05	AGTTCTATAATATAAAACA
6410	IclR family transcriptional regulator XynR	3.52E-05	GGTTATATCGATCAGAACA
6485	Hypothetical protein	2.46E-05	TCTTCTTCGCGCAAGAAAA
6530	RNA-dependent DNA polymerase	1.81E-05	AGTGCTATCACAGAGAACA
6725	ABC transporter permease	1.31E-05	TTTTCTCCGCTGATGAACA
6735	ABC transporter substrate-binding protein	5.27E-05	GGTACGCGCAGGCAGAACA
6805	Oxidoreductase	7.08E-05	TGGTATCGGCGGACGAACA
7320	Uroporphyrinogen-III C-methyltransferase	4.74E-05	TGTTCTGTTTTTAATAACT
7440	GNAT family N-acetyltranferase	9.18E-05	TGTACAACTCACCAGATCA
7740	XRE family transcriptional regulator	9.18E-05	CGTTCTCGACTGTCGAACT
8305	Recombination regulator	5.27E-05	TGCTCTCGGTGCGTGACCA
8315	Carbom storage regulator CsrA	2.39E-05	TGGTCTGGTTGTGATAACA
9200	Epimerase	2.39E-05	TGTTATCGGAAGAAGACCA
9735	Amidohydrolase	3.81E-05	TGTCCATTGTTTTAGAACA
10320	Bifunctional tetrahydrofolate synthase/dihydrofolate synthase	1.81E-05	TGTTCTGTGTCAATGAACC
10350	Histidine transport system permease protein	6.37E-06	TGCTCTGGCAGGCAGAAGA

10665	Hydrogenase formation protein	9.18E-05	TGAGCTTGCTGGATGAACA
10785	ABC transporter	2.39E-05	TGTTATTCACCAGAGAGCA
11120	Acyl-CoA dehydrogenase	4.33E-05	AATTCTCAACATAAGAACA
11345	Methyl-accepting chemotaxis protein	4.33E-05	TGATATCAAAATCAGAACA
11715	Multidrug transporter	9.18E-05	TGACCTCTGAAGCAGAACC
11825	PTS glucose transporter subunit IIBC	4.33E-05	TGTTATCAAACATTGAACA
11945	Endopeptidase	5.59E-06	TGTTCTGGCACCTAGTGCA
12540	ATP-dependent RNA helicase	7.08E-05	GGCTCTGCGCCAGAGAAAA
13225	Hypothetical protein	9.18E-05	TGGACTGGTTGCTTGAACA
13460	PLP-dependent aminotransferase family protein	3.81E-05	TGTACTGAAAAAAAGCACA
13525	Hypothetical protein	7.08E-05	TGTTTTGGCGGGTTGGACA
13740	Arabinose ABC transporter subtrate-binding protein	4.87E-05	TGTTTTTTTCTCTATAACA
13790	Dipeptide/tripeptide permease	4.33E-05	TGTCCTTCTCATAACAACA
14005	Exodeoxyribonuclease III	5.27E-05	TGGGCTGCGAGCCAGACCA
14270	Hypothetical protein	3.52E-05	TGTTTTTCAACAGAGATCA
14735	Lysogenization regulator	4.90E-05	TTATCTATTATAAAGAACA
15330	DUF480 domain-containing protein	6.36E-05	AGGTCGTCACGCCAGAACA
15415	EamA family transporter	9.18E-05	TGGCCTTCAGGCAAGAACC
15895	Hydroxylamine reductase	9.18E-05	TGTGCTTACGCTCAGGGCA
16295	ABC transporter substrate binding protein	5.59E-06	GGTTCTCAGGAGCTGAACA
16460	Hypothetical protein	2.81E-05	TGATCTGGAATATAGAGCA
16480	GlpM family protein	6.37E-06	TATTCTGCGGGGTTGAACA
16485	Pectinesterase	6.37E-06	TGTTCAACCCCGCAGAATA
16845	PTS sugar transporter subunit IIC	4.96E-05	TATTCTATTTCTTAGAAGA
17610	Dihydrodipicolinate synthase family protein	5.27E-05	TGTTCCGCTGGGCAGCACG
17850	Alpha-D-glucosyl-1-phosphate uridylyltransferase	2.81E-05	GGTTCTTTTTGAGAGAGCA
18055	Tol-pal system-associated acyl-CoA thioesterase	5.27E-05	TGTTCCGCTGGCCAGTACG
19075	NrdR trasncriptional regulator	2.39E-05	TGTTCTGCTGTTGATACCA
19130	Branched-chain amino acid transport system II carrier protein	4.33E-05	TATTCTGCTTTTTAGATCA
19145	Phosphate regulon transcriptional regulatory protein	1.81E-05	TGTGCTTTGTGCTGGAACA
19225	Type II asparaginase	7.08E-05	TGGTCAACGCGGCAGAAAA
19400	Rho-binding antiterminator	9.18E-05	CGTCCTCAGTCAGGGAACA
20180	Replicative DNA helicase	7.08E-05	TGTCTTACGGGGCCGAACA
20855	GntR family transcriptional regulator	4.50E-05	TCTTCTCAGATCAACAACA
21135	DNA-directed RNA polymerase subunit beta	2.39E-05	TGTTCTTACTGGCATAACG
21195	DedA family protein	7.13E-05	TTTTCTCGCGGGGAGAATC
21475	Acetate CoA ligase	1.31E-05	TGATCTATCGCGCAGAAAA
22275	Glucoronate isomerase	3.52E-05	TGTTCTCACAAAGATATCA
22310	Cell division protein	9.18E-05	TGCTCCGCACCATTGAACA
22700	Xylulose 5-phosphate 3-epimerase	5.27E-05	TGAACTGGACGCGGGAACA
